# Strategy for Hepatitis B and C Virus Testing Campaigns Through Web Services and Digital Advertising in Japan: Nationwide Cross-Sectional Study With Correspondence Analysis

**DOI:** 10.2196/89585

**Published:** 2026-04-02

**Authors:** Kosuke Sakai, Shoko Nakazawa, Yuko Furuya, Kota Fukai, Kei Sano, Masaaki Korenaga, Masayuki Tatemichi

**Affiliations:** 1 Department of Preventive Medicine, School of Medicine, Tokai University, 143 Shimokasuya, Isehara, 2591193, Japan, 81 463931121 ext 2613, 81 463923549; 2 Department of Ophthalmology, The Jikei University School of Medicine, Minatoku, Japan; 3 Hepatitis Information Center, The Research Center for Hepatitis and Immunology, National Institute of Global Health and Medicine, Japan Institute for Health Security, Ichikawa, Japan

**Keywords:** hepatitis B, hepatitis C, digital advertising, web services, correspondence analysis

## Abstract

**Background:**

Public awareness campaigns and testing promotion must be strengthened to eliminate infections with hepatitis B and C viruses (HBV and HCV, respectively) by 2030. Although public health campaigns using various forms of advertising are widely implemented, the most appropriate channels for viral hepatitis testing remain unclear.

**Objective:**

This study aims to identify web services and digital advertising channels appropriate for promoting HBV and HCV testing, segmented by prior testing history and the desire for hepatitis virus testing.

**Methods:**

A nationwide cross-sectional online survey of Japanese adults aged 20 to 69 years was conducted. The respondents answered questions regarding viral hepatitis testing status, routinely used web services (180 options), and exposure to digital advertising (25 options). Correspondence analysis was used to visualize relationships among testing segments, web services, and digital advertising. For individuals classified as “never having been tested and wishing to be tested,” channel-specific alignment was quantified using cosine θ. Sensitivity analyses were conducted by repeating the correspondence analysis after excluding respondents uncertain about their testing history and by fitting modified Poisson regression models with robust variance to estimate prevalence ratios and 95% CIs.

**Results:**

Of the 2000 respondents (1011 male and 989 female), 18% (n=359) reported prior HBV and HCV testing, and 22.1% (n=441) were unsure whether they had ever been tested. Web services characteristically associated with “never having been tested and wishing to be tested” included Lawson (convenience store: cosine *θ*=0.989) and Cosme (shopping: cosine *θ*=0.987). The corresponding digital advertising channels included in-store and storefront screens at Welcia (pharmacy chain: *θ*=0.994) and Lawson (cosine *θ*=0.937). Segment-specific patterns varied according to age group and sex. Sensitivity analyses excluding the unsure group showed similar patterns. Modified Poisson regression results were also consistent; for example, Lawson web service use was associated with a desire for hepatitis virus testing (prevalence ratio 1.75, 95% CI 1.22‐2.52).

**Conclusions:**

In Japan, the convenience store chain Lawson was a frequently used touchpoint, both online and offline, among individuals seeking viral hepatitis testing. Future studies are needed to determine whether implementing awareness-raising activities through Lawson can increase the uptake of testing and subsequent treatment.

## Introduction

The World Health Organization (WHO) has set the goal of eliminating hepatitis B virus (HBV) and hepatitis C virus (HCV) infections by 2030 [1]. In Japan, over 70% of patients with hepatocellular carcinoma are infected with HBV or HCV [2,3]. In 2015, approximately 2 million individuals were infected with HBV or HCV, of whom approximately 1 million were undiagnosed or untreated [4]. From 2015 to 2022, a decreasing trend in acute-onset HBV and HCV hepatitis was reported, partially attributable to strengthened surveillance systems and, in the case of HCV, to remarkable advances in treatment with direct-acting antivirals, which have dramatically reduced the disease burden [5]. However, the burden of viral hepatitis is predicted to persist until 2035, and more public health measures are required to achieve this goal [4].

In Japan, viral hepatitis testing is offered through community screening programs, health checkups, public health centers, prenatal care, and blood donations [6]. Among these, annual workplace health checkups, which cover many working-age adults, represent a particularly important opportunity for hepatitis virus testing [7]. Several companies have used this opportunity to implement HBV and HCV testing. Nevertheless, viral hepatitis screening is not part of legally required tests, resulting in only a subset of employees undergoing testing [7]. Furthermore, testing is frequently avoided due to social stigma and workplace discrimination [8]. To effectively address these challenges and enhance the response to viral hepatitis, the Ministry of Health, Labor and Welfare has formally urged employers and relevant organizations nationwide to collaborate to facilitate testing and ensure that employees who test positive receive appropriate medical treatment [9]. Hence, our research team has conducted awareness campaigns targeting top management and staff responsible for workplace health checkups within the human resources and general affairs departments [10]. To strengthen countermeasures against viral hepatitis, it is necessary to promote awareness campaigns among not only key workers but also the general population.

Recently, health awareness campaigns using websites and social media advertisements have become increasingly prevalent [11]. For example, as part of a viral hepatitis awareness campaign, educational columns and videos have been distributed via websites such as “Let’s Learn About Hepatitis” [12]. Information on the hepatitis virus has been widely disseminated to the public. However, effective health campaigns require accurately defining the target population and delivering content that is appropriately tailored to their specific attributes [13]. A scoping review of HCV media campaigns found that their effectiveness varied, emphasizing the need for customized approaches [14]. Accordingly, HCV care has been incorporated into opioid treatment programs to improve access for high-risk groups [15]. Additionally, social media strategies have been explored to identify individuals with HBV [16]. Overall, these observations indicate that designing interventions specifically for different populations is crucial for success.

Public health marketing for viral hepatitis should be tailored to a person’s testing history and results. One effective strategy to encourage more people to get tested is to guide those who have never been tested but are interested toward specific locations where they can access testing. However, there is limited knowledge regarding the most effective ways to share information about hepatitis testing with individuals who seek it.

This study aims to identify which web services and digital advertisements are most effective for promoting hepatitis testing in Japan, using groups defined by their prior testing history and interest in testing. The results of this study will inform the development of digital advertising strategies to more effectively disseminate information and promote hepatitis testing. These findings on public health advertising strategies can be applied to a range of health issues.

## Methods

### Study Design and Selection of Respondents

This nationwide cross-sectional study was conducted in August 2025 using a Japanese-language online questionnaire. Rakuten Insight Inc, a Tokyo-based web research firm, supported the survey. Panelists were users who registered through the company’s standard process and could be invited to specific surveys based on their profile information. Rakuten Insight has been used in previous peer-reviewed public health research. Respondents were recruited from the company’s registry via email and app push notifications. Invitations were randomly sent to individuals with active accounts in the past year who met the inclusion criteria as follows: aged 20 to 69 years, residing in Japan, and consenting to participate. Participants first answered screening questions; only those eligible completed the full questionnaire. Stratified sampling was carried out across 20 segments, divided by sex (male and female) and 5-year age strata, reflecting population estimates from Japan’s 2020 Population Census [17]. Surveys continued until 2000 valid responses were obtained, with target quotas set for each segment. The sample size was determined by the study budget and the maximum feasible recruitment. This study followed the STROBE (Strengthening the Reporting of Observational Studies in Epidemiology) guidelines for cross-sectional observational studies (Checklist 1) [18], and web survey reporting adhered to the CHERRIES (Checklist for Reporting Results of Internet E-Surveys) standards (Checklist 2) [19].

As this was a closed survey administered by a commercial online survey company, respondents were required to log in using their unique panel numbers before accessing the questionnaire. Once a respondent completed the survey, the questionnaire was no longer displayed. The IP addresses or cookies were not included in the survey. The survey consisted of 1 question per page, totaling 8 questions. No final review of responses was conducted.

### Assessment of HBV and HCV Testing History and Desire for Testing

Information regarding the history of hepatitis virus testing and the desire for testing was obtained using a questionnaire. The respondents were asked to indicate the statement that best described their HBV and HCV testing status: (1) having been tested and knowing the results, (2) having been tested but not knowing the results, (3) never having been tested and wishing to be tested, (4) never having been tested and not wishing to be tested, and (5) unsure if tested. In Japan, public guidance recommends that all individuals undergo hepatitis virus testing at least once in their lifetime; therefore, this study focused on ever-tested status and the current desire for testing [7].

### Assessment of Daily Used Web Services

Respondents were asked to complete multiple-choice items on regularly used web services. The survey was conducted annually by the research firm with all panel respondents. Data collected over 1 year, from November 2024 (when the questionnaire was distributed) through the completion of this survey, were analyzed. The items were synthesized from 28 genres: video streaming, video sharing, music, social networking services, search engines, news, newspapers, magazines, books, calendars, hobbies, learning, maps, points, beauty, finance, general e-commerce, electronics stores, retail, manufacturer e-commerce, online shopping, flea markets, games, cooking recipes, restaurants, food delivery, travel, and weather. Multimedia Appendix 1 lists 180 types of web services, including Google, YouTube, Amazon, Netflix, Wikipedia, Facebook, Instagram, X (formerly known as Twitter), LinkedIn, and Spotify.

### Assessment of Daily Shown Digital Advertising

Respondents were asked about their routine exposure to digital advertising by broad genres rather than brand names, such as convenience stores, supermarkets, shopping malls, drugstores, discount stores, transit media including in-car train screens, stations, bus stops, taxis, large outdoor video screens, vending machines, other noncommercial facilities, and other digital advertising (Multimedia Appendix 2).

### Other Measurements

To characterize the respondents, information was collected on sex, age, educational background, household income, marital status, and employment status. Sex was self-reported, with respondents indicating whether they were male or female. Age was determined using the age on the date of response, based on the date of birth.

Regarding their educational background, respondents were asked to select the most appropriate option from the following: middle school, high school, vocational school, junior or technical college, university, graduate school, currently enrolled, or other.

The participants were further asked to select the most appropriate option from the following choices regarding pretax household income for 2024: up to JP ¥4 million, JP ¥4.01‐6 million, JP ¥6.01‐8 million, JP ¥8.01‐10 million, JP ¥10.01‐12 million, JP ¥12.01‐15 million, and over JP ¥15 million (JP ¥159=US $1).

Marital status was defined either as married or unmarried. Regarding employment status, individuals who were currently working were asked about their main occupations and industries. Occupations were classified into 12 categories based on the Japan Standard Occupational Classification (Fifth Revision, December 2009) [20] as follows: administrative and managerial workers; professional and engineering workers; clerical workers; sales workers; service workers; security workers; agriculture, forestry, and fishery workers; manufacturing process workers; transport and machine operation workers; construction and mining workers; carrying, cleaning, packaging, and related workers; and workers unclassified by occupation [20]. Industries were classified into 20 categories based on the Japan Standard Industrial Classification: agriculture and forestry; fisheries; mining and quarrying of stone and gravel; construction; manufacturing; electricity, gas, heat, and water; information and communications; transport and postal services; wholesale and retail trade; finance and insurance; real estate and goods rental and leasing; scientific research, professional, and technical services; accommodation, food, and drink services; living-related, personal, and amusement services; education and learning support; medical, health care, and welfare; compound services; other services; government; and unclassified industries [21].

### Statistical Analysis

The numbers and percentages of respondents were compiled by sex, age, education, income, marital status, and HBV or HCV testing status. To assess selection bias in the survey, we compared the distributions of respondents’ educational backgrounds, income, occupations, industries, and residential regions with those from the 2020 Population Census [17]. The respondents’ selection status was tabulated for 180 types of web services and 25 types of digital advertisements. As all questionnaire items were set as mandatory, no data were missing.

Correspondence analysis was used to identify the web services and digital advertising appropriate for raising awareness, tailored to the history of viral hepatitis testing and the current desire for testing. Correspondence analysis is frequently used in marketing analysis [22], as it can handle large amounts of categorical data and has been employed to examine relationships between purchasing trends across numerous products and buyer attributes. In public health studies, this method has also been applied to verify the distribution of categorical data [23]. Our previous research targeting key individuals for workplace health checkups also employed this method, successfully identifying prioritized information on viral hepatitis and appropriate media for conducting campaigns [10]. Ideally, a comprehensive representation of the distribution across the 5 HBV or HCV response categories would necessitate 4 dimensions to capture the total variance (100% inertia). However, as 4D models are not practically interpretable for visualization, we selected 3 dimensions as the most suitable option for effective exploratory mapping. Statistical analyses were performed using KH Coder software (version 3.0, SCREEN Advanced System Solutions Co Ltd) [24].

To confirm the validity of the responses, a correspondence analysis was performed to visualize web service usage patterns by respondents’ sex and age. A 3D visualization of the correspondence analysis results was generated to characterize web service usage patterns associated with each viral hepatitis testing history and testing desire. To quantitatively evaluate the web service characteristics of groups willing to undergo testing (willing group), we assessed the deviation from the origin of each web service relative to the willing group using cosine θ. The same analysis was conducted for the digital advertisements. Since digital service usage patterns can differ by sex and age, the analysis was stratified by sex (male and female) and age group (20‐49 y and 50‐69 y).

Responses regarding HBV or HCV testing history and intention were classified into 5 mutually exclusive categories: tested and known result, tested but unknown result, never tested and willing to be tested, never tested and unwilling to be tested, and unsure if previously tested. The category “unsure if tested” was treated as an independent variable, as uncertainty about prior testing was considered meaningful for public health communication and may reflect distinct factors, such as limited recall and incomplete communication of test results.

During the sensitivity analysis, we excluded respondents who selected “unsure if tested” and subsequently performed correspondence analysis to identify web services and digital advertising channels associated with the willing group. Additionally, we fitted a series of modified Poisson regression models with robust variance, treating each of the web services selected by at least 5% (100/2000) and each of the digital advertisements as binary outcomes. Although the correspondence analysis used all web services, the multivariate regression analysis included only selected web services to adjust for covariates. The main independent variable was the desire for viral hepatitis testing (never tested but want to be tested vs all other responses). All models were adjusted for sex, age, household income, educational background (less than university vs university or higher), and marital status. Models with web services as outcomes were further adjusted for the total number of web services selected (0‐180); models with digital advertisements as outcomes were further adjusted for the total number of advertisements selected (0‐25). Age, sex, household income, educational attainment, and marital status were included as covariates as they likely influence prior testing history and desire for hepatitis virus testing [25]. Moreover, the total number of selected web services or digital advertising channels was included, as respondents demonstrating a higher overall propensity to choose multiple digital options are, by definition, more likely to exhibit positive results for any specific outcome. The detailed variable definitions and coding schemes (web services and digital advertising channels) are presented in Multimedia Appendix 3. For each outcome, we estimated prevalence ratios (PRs) and 95% CIs for the desire for viral hepatitis testing, with a 2-sided significance level of 5%. The statistical software used was R (version 4.4.2; R Foundation for Statistical Computing) [26].

### Ethical Considerations

This study was approved by the research ethics committee of the Tokai University School of Medicine (23R039). Informed consent was obtained from all participants before they responded to the questionnaire. The consent screen outlined the study’s purpose, procedures, voluntary participation, and the process for withdrawing consent. Respondents were allowed to withdraw their consent up to 3 months after survey completion. To protect privacy, Rakuten Insight removed all personally identifiable information before data delivery. These measures were designed to minimize the risk of information leakage. Participants earned panel points based on the provider’s standard incentive system: those who completed the survey received 5 points, each equivalent to JP ¥1 (US $0.6), which were redeemable within the provider’s partner network.

## Results

### Participants

**Figure 1.**
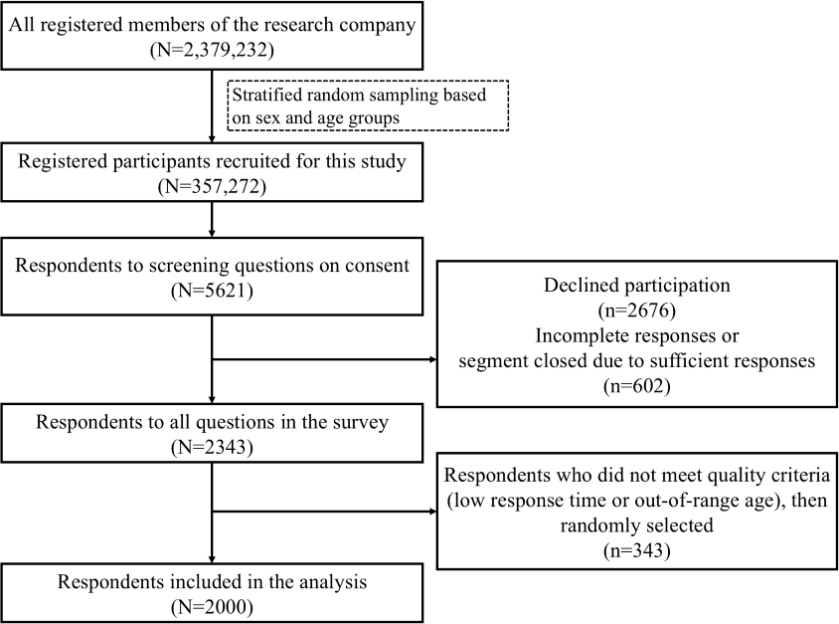
Flowchart of participant selection for analysis.

Of the 2,379,232 registered individuals in the survey panel, 357,272 were invited to participate in the study via email and push notifications. Of these, 5621 responded to the screening question (Figure 1). A total of 3278 individuals were excluded due to declining participation (n=2676), incomplete responses, or reaching quota targets (n=602). Among the 2343 individuals who answered all questions, 2000 were selected for analysis using the following process. First, respondents were excluded if their response times were below a threshold defined as the median response time multiplied by a device factor (0.3 for PC and 0.2 for smartphone). Subsequently, a random number was assigned to each valid sample using the RAND function in Microsoft Excel. The samples were ranked in ascending order according to assigned random numbers, and the top 2000 were selected for inclusion in the final dataset. The invitation-to-screening proportion was 1.6% (5621/357,272), while the screening-to-analysis proportion was 85.4% (2000/2343).

### Characteristics of the Respondents

Responses were obtained from 1011 male and 989 female participants (Table 1). Most respondents (484/2000, 24.2%) were aged 50 to 59 years. The educational background was university or graduate school for 56.3% (569/1011) of the male and 37.6% (372/989) of the female participants. A household income of less than JP ¥4 million was the most common, accounting for 31.4% (317/1011) of male and 41.2% (407/989) of female participants. Regarding marital status, 48.4% (489/1011) of male and 52.1% (515/989) of female participants were married. The percentages of respondents who had been tested for HBV or HCV and knew their results were 14.4% (146/1011) among male and 18.7% (185/989) among female participants. The percentage of individuals who were never tested but wanted to be tested was 26% (263/1011) among male and 23.4% (231/989) among female participants.

To assess selection bias, we compared the distributions of educational background (Multimedia Appendix 4), household income (Multimedia Appendix 5), occupation and industry (Multimedia Appendix 6), and residential region (Multimedia Appendix 7) with those from the 2020 Population Census of Japan.

Regarding educational attainment, national statistics indicate that 27.8% of male and 17.9% of female participants hold university degrees, whereas in this study, 50.1% of male and 34.8% of female participants hold university degrees.

Regarding household income, the census showed that 48.9% of households earned less than JP ¥4 million, whereas only 36.2% of respondents in this study were in this income bracket. Among male participants, administrative and managerial workers accounted for 2.1% in the census and 14.5% in this study, sales workers accounted for 12.4% in the census and 5.8% in this study, and manufacturing process workers accounted for 13.4% in the census and 6.3% in this study. The distribution of industries among the respondents in this study was similar to that in the census.

Regarding residential regions, participants were overrepresented in Tokyo (male: +2.41% points; female: +2.7% points), Kanagawa (male: +2.24% points; female: +2.47% points), and Osaka (male: +2.13% points; female: +0.70% points) compared with the national population distribution.

**Table 1.** Respondent characteristics.

Characteristics	All (N=2000), n (%)	Male (n=1011), n (%)	Female (n=989), n (%)
Age (y)			
20‐29	339 (17)	175 (17.3)	164 (16.6)
30‐39	351 (17.6)	180 (17.8)	171 (17.3)
40‐49	433 (21.7)	219 (21.7)	214 (21.6)
50‐59	484 (24.2)	244 (24.1)	240 (24.3)
60‐69	393 (19.7)	193 (19.1)	200 (20.2)
Educational background			
Middle or high school	534 (26.7)	254 (25.1)	280 (28.3)
Vocational school, junior college, or technical college	434 (21.7)	130 (12.9)	304 (30.7)
University or graduate school	941 (47.1)	569 (56.3)	372 (37.6)
Currently enrolled	73 (3.7)	47 (4.6)	26 (2.6)
Other	18 (0.9)	11 (1.1)	7 (0.7)
Household income (JP ¥^a^)			
≤4 million	724 (36.2)	317 (31.4)	407 (41.2)
4.01‐6 million	414 (20.7)	205 (20.3)	209 (21.1)
6.01‐8 million	358 (17.9)	192 (19)	166 (16.8)
8.01‐10 million	236 (11.8)	138 (13.6)	98 (9.9)
10.01‐12 million	133 (6.7)	79 (7.8)	54 (5.5)
12.01‐15 million	73 (3.7)	42 (4.2)	31 (3.1)
>15 million	62 (3.1)	38 (3.8)	24 (2.4)
Marital status			
Married	1004 (50.2)	489 (48.4)	515 (52.1)
Unmarried	996 (49.8)	522 (51.6)	474 (47.9)
History of HBV^b^ and HCV^c^ testing and current desire for testing			
Tested and known result (known)	331 (16.6)	146 (14.4)	185 (18.7)
Tested but unknown result (unknown)	28 (1.4)	19 (1.9)	9 (0.9)
Never tested, want to be (willing)	494 (24.7)	263 (26)	231 (23.4)
Never tested, do not want to be (unwilling)	706 (35.3)	358 (35.4)	348 (35.2)
Unsure if tested (unsure)	441 (22.1)	225 (22.3)	216 (21.8)

a JP ¥159=US $1.

b HBV: hepatitis B virus.

c HCV: hepatitis C virus

### Characteristics of Web Services and Digital Advertising

The top 50 selected web services are presented in Multimedia Appendix 8. The top 10 selected services were the general e-commerce company Rakuten Ichiba (n=863), Google (n=740), Amazon (n=690), YouTube (n=669), the search engine Yahoo! Japan (n=658), Yahoo! News (n=624), Google Maps (n=589), Yahoo! Weather & Disaster (n=503), the travel reservation site Rakuten Travel (n=455), and the restaurant review site Tabelog (n=420).

The ranking of the 25 types of digital advertising is presented in Multimedia Appendix 9. The most frequent responses were “Nothing particular” at 53.1% (1062/2000), “FamilyMart in-store and storefront digital advertising” at 18.3% (365/2000), “Digital advertising inside trains” at 17.9% (358/2000), “Digital advertising at stations and inside station facilities” at 17.3% (346/2000), and “Seven-Eleven in-store and storefront digital advertising” at 16.1% (322/2000).

### Correspondence Analysis Between Respondent Characteristics and Web Services or Digital Advertisements

The results of the correspondence analysis between sex and web services are presented in Multimedia Appendix 10. The specific web services used by male participants included internet technology services such as IT media and DMM.com, as well as newspaper sites such as Nikkei and Yomiuri. Among female participants, the most common services were cosmetic services and cooking recipe sites.

The results of the correspondence analysis between age groups and web services are shown in Multimedia Appendix 11. In their 20s, Uber Eats (food delivery), TikTok (video sharing), Spotify (music), and gaming services were prominent; in their 30s and 40s, Cosme (cosmetic shopping), Rakuten Fashion (general e-commerce), HOT PEPPER Beauty (beauty salon booking and review site), and Cookpad (recipe sharing and cooking website); in their 50s, Yahoo sites (search engine and relevant services) and Facebook; and in their 60s, Japanet (TV shopping), MSN Japan (online news and portal site), and Belluna (mail-order and online shopping site).

### Correspondence Analysis Between HBV and HCV Testing Characteristics and Web Services or Digital Advertising

**Figure 2.**
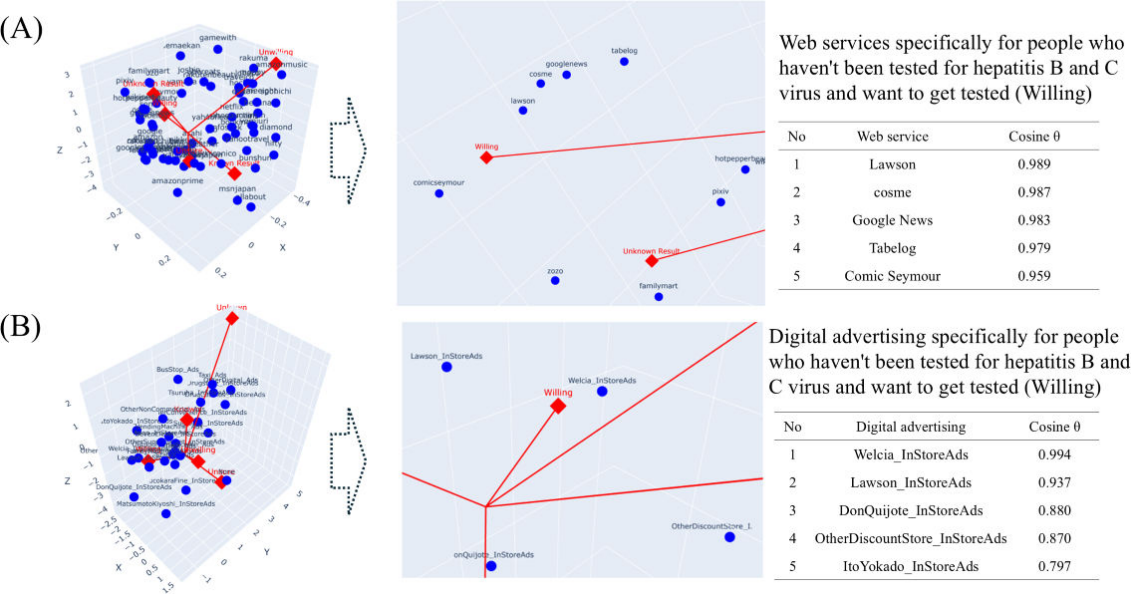
Associations of viral testing history and the desire for testing with (A) web services and (B) digital advertising.

Figure 2 shows the 3 dimensions: correspondence analysis results for the respondents’ HBV and HCV testing statuses, the 60 web services farthest from the origin, and all 25 digital advertisements. Web services specific to people who had not been tested and wanted to be tested were Lawson (convenience store) with a cosine θ of 0.989 and Cosme (cosmetic shopping) with a cosine θ of 0.987. Digital advertising targeted at individuals who had not been tested and wanted to be tested included in-store and storefront advertising at Welcia (pharmacy chain; cosine *θ*=0.994) and Lawson (convenience store; cosine *θ*=0.937).

**Figure 3.**
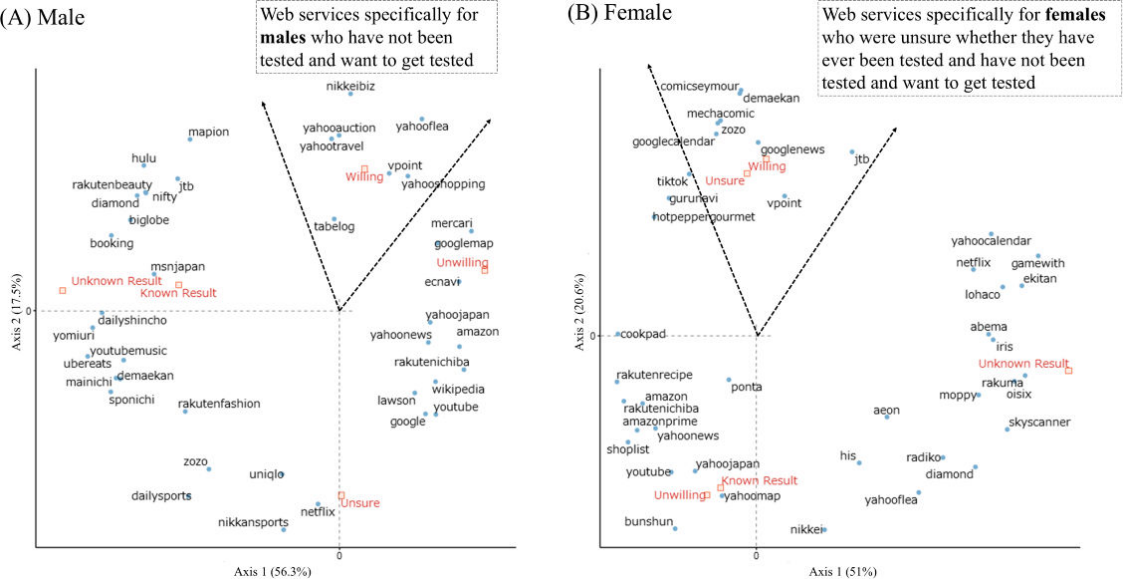
Relationship between hepatitis B virus (HBV) or hepatitis C virus (HCV) screening experience and web services by sex. (A) Results for male participants. (B) Results for female participants.

The results of the correspondence analysis between HBV and HCV testing status and web services by sex (male and female) and age group (<50 y or >50 y) are shown in Figures3  4, respectively. Depending on sex and age group, web services specific to individuals who had not been tested and who wanted to be tested were identified. Among individuals who intended to undergo testing, the most frequently used were Nikkei Biz (news media) and Yahoo-related services (search engine) for male participants, Demae-can (food delivery) and e-book platforms (Comic Seymour and Mecha Comic) for female participants, Google-related services for those aged 20 to 49 years, and Zozotown (fashion e-commerce site) and Booking.com (hotel booking site) for those aged 50 to 69 years. In the correspondence analysis, the first 2 dimensions explained most of the variability in the distribution of web services: among male participants, 73.8% of the total inertia (56.3% on axis 1 and 17.5% on axis 2), and among female participants, 71.6% (51% on axis 1 and 20.6% on axis 2). For participants aged 20 to 49 years, the first 2 dimensions accounted for 82.1% of total inertia (70.5% on axis 1 and 11.6% on axis 2); for those aged 50 to 69 years, they accounted for 68% (40.7% on axis 1 and 27.3% on axis 2).

**Figure 4.**
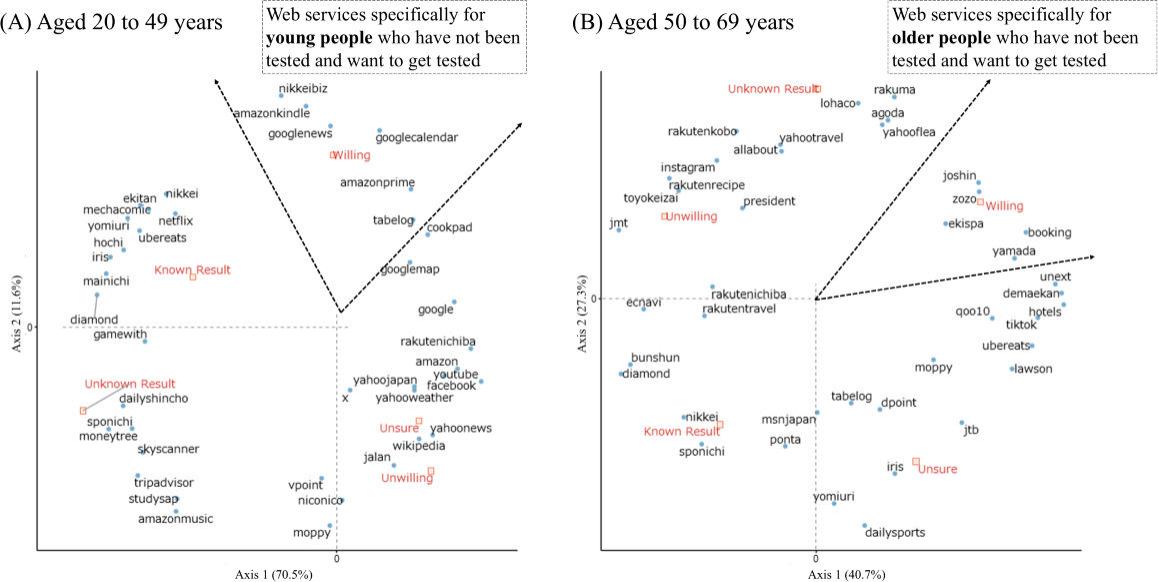
Relationship between hepatitis B virus (HBV) or hepatitis C virus (HCV) screening experience and web services by age groups. (A) Results for respondents aged 20 to 49 years. (B) Results for respondents aged 50 to 69 years.

### Sensitivity Analysis

The findings from the sensitivity analysis of web services using correspondence analysis are detailed in Multimedia Appendix 12. Web services identified as particularly relevant for individuals who had not been tested but wanted to be included are Google News (cosine *θ*=0.98), Zozotown—a fashion e-commerce platform—(cosine *θ*=0.96), Nikkei Biz (news media; cosine *θ*=0.94), Comic Seymour (e-book platform; cosine *θ*=0.92), and Lawson (convenience store; cosine *θ*=0.90).

Similarly, the outcomes of the sensitivity analysis of digital advertising channels indicated that Lawson in-store and storefront advertisements (cosine *θ*=0.96), as well as Matsumoto Kiyoshi in-store and storefront advertisements (cosine *θ*=0.92), were specifically associated with individuals who had not been tested but wanted to be (Multimedia Appendix 13).

The results of the sensitivity analysis of the web services using a modified Poisson regression revealed a significant association for Lawson (Multimedia Appendix 14). The highest PRs for the desire for hepatitis testing were observed for Lawson (PR 1.75, 95% CI 1.22‐2.52), Zozotown (PR 1.67, 95% CI 1.18‐2.36), Google Calendar (PR 1.63, 95% CI 1.21‐2.20), and Google News (PR 1.61, 95% CI 1.25‐2.07).

The sensitivity analysis of digital advertising channels, using modified Poisson regression, demonstrated that the highest PRs were observed for in-store advertisements at other discount stores (PR 2.64, 95% CI 1.51‐4.59), Ito Yokado (PR 1.72, 95% CI 1.13‐2.63), DonQuijote (PR 1.72, 95% CI 1.18‐2.51), Welcia (PR 1.71, 95% CI 1.13‐2.59), and Lawson (PR 1.49, 95% CI 1.19‐1.87; Multimedia Appendix 15).

## Discussion

### Principal Findings

We identified web services and digital advertisements for use in HBV and HCV campaign channels, based on testing history and expressed interest in testing. Individuals who had never been tested for hepatitis but wanted to be tested were regularly exposed to the website and in-store digital advertisements of Lawson, one of the most famous convenience store chains. Segmenting the target population by sex or age group revealed distinct preferences for web services. These observations suggest that effective digital public health campaigns should account for specific attributes of the target population, including their testing history and interests, when determining appropriate communication channels.

### Comparison With Prior Work

Lawson might be crucial in conducting viral hepatitis awareness campaigns, as individuals interested in HBV and HCV testing use Lawson’s web services and digital advertisements. In 2013, Lawson collaborated with local medical and public health organizations to distribute application forms for free hepatitis testing at selected stores in Saitama Prefecture, illustrating the potential of convenience store chains as access points for hepatitis screening campaigns [27]. Convenience stores are gaining attention as venues for health promotion. For example, initiatives have been undertaken to improve access to healthy foods by stocking them in these stores [28,29]. Convenience stores are key locations within the community, and staff education has been implemented to strengthen the community network for dementia [30]. These initiatives focused on location characteristics, such as the presence of unhealthy food and local staff members. The results of this study enable us to consider intervention settings from different perspectives by designing interventions tailored to the characteristics of visiting customers.

In public health communication, it is preferable to tailor information dissemination to the characteristics and interests of the target audience [31]. As advertising is expensive, cost-effective campaigns can be implemented by targeting those who have never undergone an examination but are willing to be tested. Several interventions have been tailored to specific target groups based on their characteristics [32]. An educational intervention using an app to provide information about HBV screening in their native language to Asian Americans resulted in increased consultations with health care providers and subsequent testing [33]. In the United Kingdom, a combination intervention of sending tailored letters to encourage high-risk and untreated patients with HCV to seek care, training staff, and implementing awareness-raising notices increased HCV testing and treatment, demonstrating good cost-effectiveness [34]. In an intervention in New York, a multidisciplinary team implemented efforts to remove barriers to care for low-income individuals, the homeless, and drug users who needed support, leading many to undergo HCV testing and complete treatment [35]. Consistent with these results, community-based interventions at convenience stores may be a promising approach.

When developing advertising strategies for public health, it is important to consider the sex and age of the target population. The results of this study revealed distinct patterns in web service usage and digital advertising exposure by sex and age. Therefore, stratified by sex and age, the characteristic channels differed according to viral hepatitis testing status. Viral hepatitis testing is recommended at least once in a lifetime, regardless of sex or age [36]. For this awareness campaign, it was preferable to use the analysis results based on the entire target population. When focusing on specific age groups or sex, awareness strategies should be developed based on analysis results adjusted for the characteristics of that group. For example, when implementing HBV awareness campaigns targeting pregnant female individuals, awareness campaigns should be developed by referencing our results in female participants.

Fewer respondents than expected reported having been tested for viral hepatitis. In Japan, it is estimated that more than half of the population has been tested for viral hepatitis [6], far exceeding the 18% testing rate in this study; however, an additional 22.1% were unsure of whether they had been tested. A study investigating Japanese people’s knowledge of how viral hepatitis testing is performed reported that 93% of respondents identified blood tests, whereas only 7% mentioned urine tests [37]. In addition, physicians do not inform patients of negative results. A survey targeting ophthalmologists who frequently conducted viral hepatitis tests before surgery revealed that approximately 40% did not explain the results to patients when the test was negative [38]. Even when negative results were verbally communicated to patients, only 42% recalled them 1 year later, leading to the recommendation of using negative cards—written records certifying that the individual tested negative for HBV and HCV—for communicating results [39]. Negative cards are expected to enable information sharing with other health care professionals and to avoid duplicate viral hepatitis testing.

This study neither implemented nor evaluated specific public health awareness campaigns. Instead, we present a methodological case study illustrating how correspondence analysis can inform the development of public health strategies that leverage web services and digital advertising. Medical professionals have used correspondence analysis to address critical issues through dimension reduction, data simplification, clustering, and classification [40]. This study visualized the distributions of 180 web services and 25 digital advertisements. Correspondence analysis has been used to clarify the relationship between age and headache type, to identify associations between personality type and various diagnoses [41], and to clarify the key challenges prioritized by each profession when pursuing innovation in public hospitals [42]. On the basis of these examples, correspondence analysis may be useful as an exploratory tool for examining and planning public health advertising strategies. A sensitivity analysis using multivariate models adjusted for respondent characteristics yielded patterns similar to those observed in the main correspondence analysis. This consistency indicates that the study’s findings are robust to potential confounding from participant attributes.

In this study, respondents tended to have higher educational attainment, higher household income, and a greater proportion of managerial occupations than the working population estimated from the 2020 Population Census. This is consistent with prior research showing that online surveys often include individuals with higher education and income levels than those in national statistics [43,44]. Higher socioeconomic status has been associated with better health literacy, which may explain the interest in hepatitis testing [45]. Moreover, because web service use was assessed among individuals who chose to participate in an online survey, respondents might be more familiar with online services. Thus, the level of web service use in this study might be higher than that in the general population, and our findings reflect patterns among digitally engaged individuals.

### Limitations

This study has certain limitations. First, potential selection bias warrants consideration. Compared with national statistics, our study included a higher proportion of individuals with university-level education, lower annual income, and a higher proportion in managerial or professional roles than in production-related occupations. Furthermore, respondents were more likely to reside in urban areas. Given that increased educational attainment, income, and occupational status may correlate with heightened health awareness and interest in viral hepatitis testing, the observed associations may not be generalizable to the broader population. Thus, the findings must be interpreted with caution when extending them beyond digitally engaged adults. Future studies should address this issue by using non–internet-based recruitment methods to enhance external validity.

Second, measurement biases regarding web services and digital advertising exist. To mitigate this effect, we conducted a correspondence analysis of web service usage by sex and age, revealing patterns consistent with real-world behaviors—such as female individuals preferring cosmetic sites and younger individuals favoring TikTok. Subsequent research should seek to collect objective data regarding web service use.

Third, recall bias may have influenced the reporting of HBV or HCV testing history. Notably, 22.1% of respondents indicated uncertainty about previous testing, which may have led to misclassification of past testing status. Additionally, exposure misclassification may have occurred because digital advertising exposure was self-reported and may not accurately reflect media saturation or individual engagement with advertisements.

Finally, we were unable to verify the effectiveness of advertising campaigns in changing behavior. Further investigation is necessary to determine whether interventions that incorporate appropriate web services and digital advertising can facilitate viral hepatitis testing, detection, and treatment.

### Conclusions

This study identified distinct channel-specific patterns in the promotion of viral hepatitis testing in Japan. Lawson consistently served as a primary point of contact across web services and digital advertising among respondents who had never been tested but expressed interest in testing. Subgroup analyses revealed differences by sex and age, while sensitivity analyses confirmed comparable overall trends. These results support the development of targeted digital communication strategies and offer a practical framework for designing effective public health campaigns for HBV or HCV testing.

## Supplementary material

10.2196/89585 Multimedia Appendix 1 List of 180 types of web services.

10.2196/89585 Multimedia Appendix 2 List of 25 types of digital advertising.

10.2196/89585 Multimedia Appendix 3 Variables used in modified Poisson regression models for digital exposure and desire for hepatitis virus testing.

10.2196/89585 Multimedia Appendix 4 Distribution of educational background in this study and the Census 2020.

10.2196/89585 Multimedia Appendix 5 Distribution of household income in this study and the Census 2020.

10.2196/89585 Multimedia Appendix 6 Distribution of occupation and industry in this study and the census 2020.

10.2196/89585 Multimedia Appendix 7 Distribution of residential regions in this study and the 2020 Population Census.

10.2196/89585 Multimedia Appendix 8 The top-selected 50 web services.

10.2196/89585 Multimedia Appendix 9 The selection of 25 digital advertising.

10.2196/89585 Multimedia Appendix 10 Correspondence analysis between sex and web services.

10.2196/89585 Multimedia Appendix 11 Correspondence analysis between age groups and web services.

10.2196/89585 Multimedia Appendix 12 Sensitivity analysis of web services based on 3D correspondence analysis.

10.2196/89585 Multimedia Appendix 13 Sensitivity analysis of digital advertising channels based on 3D correspondence analysis.

10.2196/89585 Multimedia Appendix 14 Associations between desire for hepatitis virus testing and use of individual web services.

10.2196/89585 Multimedia Appendix 15 Associations between desire for hepatitis virus testing and exposure to individual digital advertising channels.

10.2196/89585 Checklist 1 STROBE checklist.

10.2196/89585 Checklist 2 CHERRIES checklist.

## Abbreviations

CHERRIES: Checklist for Reporting Results of Internet E-Surveys
HBV: hepatitis B virus
HCV: hepatitis C virus
PR: prevalence ratio
STROBE: Strengthening the Reporting of Observational Studies in Epidemiology
WHO: World Health Organization

## References

[1] (2022). Global health sector strategies on, respectively, HIV, viral hepatitis and sexually transmitted infections for the period 2022-2030. https://iris.who.int/server/api/core/bitstreams/6b18ac34-f56a-466e-9660-b5653cb52ef0/content.

[2] Ikeda M, Mitsunaga S, Shimizu S (2013). Current status of hepatocellular carcinoma in Japan. Chin Clin Oncol.

[3] Nakano M, Yatsuhashi H, Bekki S (2022). Trends in hepatocellular carcinoma incident cases in Japan between 1996 and 2019. Sci Rep.

[4] Tanaka J, Kurisu A, Ohara M (2022). Burden of chronic hepatitis B and C infections in 2015 and future trends in Japan: a simulation study. Lancet Reg Health West Pac.

[5] Okushin K, Kanto T, Korenaga M (2025). Real‐world trends in acute viral hepatitis in Japan: a nationwide questionnaire‐based survey. Hepatol Res.

[6] Korenaga M, Kanto T (2021). Testing, diagnosis of viral hepatitis, and the follow-up policy in Japan. Glob Health Med.

[7] Tatemichi M, Furuya H, Nagahama S (2020). A nationwide cross-sectional survey on hepatitis B and C screening among workers in Japan. Sci Rep.

[8] Sasaki N, Wada K, Smith DR, Wang G, Ohta H, Shibuya A (2014). Hepatitis screening in Japanese individuals of working age and prejudice against infected persons in the workplace. J Occup Health.

[9] (2023). Request for cooperation regarding measures against viral hepatitis in the workplace [Report in Japanese]. https://www.mhlw.go.jp/content/10900000/001076013.pdf.

[10] Sakai K, Nakazawa S, Fukai K, Furuya Y, Korenaga M, Tatemichi M (2025). Marketing strategies for promoting workplace hepatitis B and C virus testing: a cross-sectional study using correspondence analysis in Japan. Front Public Health.

[11] An J, Kwak H, Qureshi HM, Weber I (2021). Precision public health campaign: delivering persuasive messages to relevant segments through targeted advertisements on social media. JMIR Form Res.

[12] Takeuchi Y, Ohara M, Kanto T (2021). Nationwide awareness-raising program for viral hepatitis in Japan: the “*Shitte kan-en*” project. Glob Health Med.

[13] Dietrich T, Dietrich T, Rundle-Thiele S, Kubacki K (2017). Segmentation in Social Marketing.

[14] Etoori D, Desai M, Mandal S, Rosenberg W, Sabin CA (2023). A scoping review of media campaign strategies used to reach populations living with or at high risk for hepatitis C in high income countries to inform future national campaigns in the United Kingdom. BMC Infect Dis.

[15] Talal AH, Dharia A, Markatou M (2025). Facilitated telemedicine as a patient-centered, sociotechnical intervention to integrate hepatitis C treatment into opioid treatment programs and overcome the digital divide among underserved populations: qualitative study. JMIR Public Health Surveill.

[16] Willem T, Zimmermann BM, Matthes N, Rost M, Buyx A (2024). Acceptance of social media recruitment for clinical studies among patients with hepatitis B: mixed methods study. J Med Internet Res.

[17] (2020). Population census [Article in Japanese]. e‑Stat.

[18] von Elm E, Altman DG, Egger M (2007). The Strengthening the Reporting of Observational Studies in Epidemiology (STROBE) statement: guidelines for reporting observational studies. Ann Intern Med.

[19] Eysenbach G (2004). Improving the quality of web surveys: the Checklist for Reporting Results of Internet E-Surveys (CHERRIES). J Med Internet Res.

[20] (2009). Japan Standard Occupational Classification (rev. 5th, December 2009) structure and explanatory notes [Website in Japanese]. Ministry of Internal Affairs and Communications.

[21] (2013). Japan Standard Industrial Classification (rev. 13, October 2013) the underlying principles of the classification [Website in Japanese]. Ministry of Internal Affairs and Communications.

[22] Beh EJ, Lombardo R (2014). Correspondence Analysis: Theory, Practice and New Strategies.

[23] Costa PS, Santos NC, Cunha P, Cotter J, Sousa N (2013). The use of multiple correspondence analysis to explore associations between categories of qualitative variables in healthy ageing. J Aging Res.

[24] Higuchi K (2017). New quantitative text analytical method and KH Coder software. Jpn Sociol Rev.

[25] Hajek A, Kretzler B, König HH (2021). Determinants of healthcare use based on the Andersen model: a systematic review of longitudinal studies. Healthcare (Basel).

[26] Core Team R (2024). R: a language and environment for statistical computing. R Foundation for Statistical Computing.

[27] News release. Lawson, Inc.

[28] Haboush-Deloye AL, Knight MA, Bungum N, Spendlove S (2023). Healthy foods in convenience stores: benefits, barriers, and best practices. Health Promot Pract.

[29] Jernigan VBB, Williams M, Wetherill M (2018). Using community-based participatory research to develop healthy retail strategies in Native American–owned convenience stores: the THRIVE study. Prev Med Rep.

[30] Igarashi A, Matsumoto H, Takaoka M, Kugai H, Suzuki M, Yamamoto-Mitani N (2020). Educational program for promoting collaboration between community care professionals and convenience stores. J Appl Gerontol.

[31] Schmid KL, Rivers SE, Latimer AE, Salovey P (2008). Targeting or tailoring? Maximizing resources to create effective health communications. Mark Health Serv.

[32] Rajkumar V, McCausland K, Lobo R (2022). A rapid review of interventions to increase hepatitis B testing, treatment, and monitoring among migrants living in Australia. Int J Environ Res Public Health.

[33] Khalili M, Kim NJ, Tsoh JY (2022). Health Within Reach-a patient-centered intervention to increase hepatitis B screening among Asian Americans: a randomized clinical trial. J Gen Intern Med.

[34] Roberts K, Macleod J, Metcalfe C (2020). Cost effectiveness of an intervention to increase uptake of hepatitis C virus testing and treatment (HepCATT): cluster randomised controlled trial in primary care. BMJ.

[35] Ford MM, Johnson N, Desai P, Rude E, Laraque F (2017). From care to cure: demonstrating a model of clinical patient navigation for hepatitis C care and treatment in high-need patients. Clin Infect Dis.

[36] (2011). Basic guidelines for promotion of control measures for hepatitis. Ministry of Health, Labour and Welfare.

[37] Masuda S, Hidaka I, Nakano S (2025). Knowledge enlightenment about viral hepatitis using a quiz at an awareness-raising event. Kanzo.

[38] Ohara M, Ide T, Inoue J (2025). Follow-up survey on hepatitis virus testing among ophthalmologists. Kanzo.

[39] Aida M, Ikegami T, Korenaga M (2023). Methods of communicating negative results of hepatitis virus tests. Kanzo.

[40] Žlahtič B, Kokol P, Blažun Vošner H, Završnik J (2024). The role of correspondence analysis in medical research. Front Public Health.

[41] Greenacre M (1992). Correspondence analysis in medical research. Stat Methods Med Res.

[42] Jończyk JA, Olszewska AM (2016). The use of correspondence analysis in assessing the antecedents of innovativeness in public hospitals. Stud Log Gramm Rhetor.

[43] Tsuboi S, Yoshida H, Ae R, Kojo T, Nakamura Y, Kitamura K (2015). Selection bias of internet panel surveys: a comparison with a paper-based survey and national governmental statistics in Japan. Asia Pac J Public Health.

[44] Szolnoki G, Hoffmann D (2013). Online, face-to-face and telephone surveys—comparing different sampling methods in wine consumer research. Wine Econ Policy.

[45] Kagamimori S, Gaina A, Nasermoaddeli A (2009). Socioeconomic status and health in the Japanese population. Soc Sci Med.

[46] Introducing GPT‑5.2. OpenAI.

