# Supplementary material for: Strategy for Hepatitis B and C Virus Testing Campaigns Through Web Services and Digital Advertising in Japan: Nationwide Cross-Sectional Study With Correspondence Analysis
Source: J Med Internet Res. 2026 Apr 2;28:e89585. doi: 10.2196/89585 (PMC13046096; doi:10.2196/89585)
Supplement: Multimedia Appendix 1 [file jmir-v28-e89585-s001.docx]

# Multimedia Appendix 1. List of 180 types of web services

| No | Genre | Site Name (English) | Site Name (Japanese) | Variable name  (analysis code) |
| --- | --- | --- | --- | --- |
| 1 | Video streaming | Amazon Prime Video | Amazon Prime Video | amazonprime |
| 2 | Video streaming | Netflix | Netflix（ネットフリックス） | netflix |
| 3 | Video streaming | Hulu | Hulu（フールー） | hulu |
| 4 | Video streaming | U-NEXT | U-NEXT（ユーネクスト） | unext |
| 5 | Video streaming | ABEMA | ABEMA（アベマ） | abema |
| 6 | Video streaming | Disney+ | Disney+ (ディズニープラス) | disneyplus |
| 7 | Video streaming | d Anime Store | dアニメストア | danime |
| 8 | Video streaming | Niconico / Niconico Returns | ニコニコ動画・帰ってきたニコニコ | niconico |
| 9 | Video streaming | DAZN | DAZN | dazn |
| 10 | Video streaming | FOD | FOD | fod |
| 11 | Video streaming | Lemio | Lemio | lemio |
| 12 | Video streaming | TERASA | TERASA | terasa |
| 13 | Video streaming | NTV TADA | 日テレTADA | nitteretada |
| 14 | Video streaming | TBS FREE | TBSFREE | tbsfee |
| 15 | Video streaming | DMM TV | DMM TV | dmmtv |
| 16 | Video streaming | TVer | TVer | tver |
| 17 | Video shareing | YouTube | YouTube | youtube |
| 18 | Video shareing | TikTok | TikTok | tiktok |
| 19 | Video shareing | BIGO LIVE | BIGO LIVE | bigolive |
| 20 | Video shareing | 17LIVE | 17LIVE | ichinanalive |
| 21 | Video shareing | Pococha | Pococha（ポコチャ） | pococha |
| 22 | Video shareing | REALITY | REALITY | reality |
| 23 | Music | Apple Music | Apple Music | applemusic |
| 24 | Music | YouTube Music | YouTube Music | youtubemusic |
| 25 | Music | Spotify | Spotify | spotify |
| 26 | Music | LINE MUSIC | LINE MUSIC | linemusic |
| 27 | Music | Amazon Music | Amazon Music | amazonmusic |
| 28 | Music | radiko | radiko（ラジコ） | radiko |
| 29 | Social networking service | Facebook | Facebook | facebook |
| 30 | Social networking service | Instagram | Instagram | instagram |
| 31 | Social networking service | LinkedIn | Linkedin | linkedin |
| 32 | Social networking service | LINE | LINE | Line |
| 33 | Social networking service | X (formerly Twitter) | X（旧Twitter） | x |
| 34 | Social networking service | Threads | Threads（スレッズ） | threads |
| 35 | Search engine | Google | Google | google |
| 36 | Search engine | Yahoo! Japan | Yahoo!Japan | yahoojapan |
| 37 | Search engine | MSN Japan | MSN Japan | msnjapan |
| 38 | Search engine | goo | goo | goo |
| 39 | Search engine | BIGLOBE | BIGLOBE | biglobe |
| 40 | Search engine | Infoseek | Infoseek | infoseek |
| 41 | Search engine | @nifty | ＠nifty | nifty |
| 42 | News | ITmedia | ITmedia | itmedia |
| 43 | News | iza | iza | iza |
| 44 | News | J-CAST | J-CAST | jcast |
| 45 | News | All About | All About | allabout |
| 46 | News | zakzak | zakzak | zakzak |
| 47 | News | Jiji.com / Jiji Press News Next | 時事ドットコム／時事通信ニュースnext | jiji |
| 48 | News | Google News | Google ニュース | googlenews |
| 49 | News | Yahoo! News | Yahoo!ニュース | yahoonews |
| 50 | News | Gunosy | グノシー | gunosy |
| 51 | News | NewsPicks | NewsPicks | newspicks |
| 52 | News | Wikipedia | Wikipedia | wikipedia |
| 53 | Newspapers | Yomiuri Shimbun Online | 読売新聞オンライン | yomiuri |
| 54 | Newspapers | Asahi Shimbun Digital | 朝日新聞デジタル | asahi |
| 55 | Newspapers | Mainichi Shimbun Digital | 毎日新聞デジタル | mainichi |
| 56 | Newspapers | Sankei News | 産経ニュース | sankei |
| 57 | Newspapers | Nikkei (Digital Edition) | 日本経済新聞（電子版） | nikkei |
| 58 | Newspapers | Nikkan Sports | ニッカンスポーツ・コム | nikkansports |
| 59 | Newspapers | Sanspo | サンスポ | sanspo |
| 60 | Newspapers | Sponichi Annex | スポニチ Sponichi Annex/スポニチ | sponichi |
| 61 | Newspapers | Daily Sports | デイリー/デイリースポーツ | dailysports |
| 62 | Newspapers | Sports Hochi | スポーツ報知 | hochi |
| 63 | Magazine | Diamond Online | ダイヤモンド・オンライン | diamond |
| 64 | Magazine | Toyo Keizai Online | 東洋経済オンライン | toyokeizai |
| 65 | Magazine | PRESIDENT Online | PRESIDENT Online | president |
| 66 | Magazine | Nikkei Business Digital Edition | 日経ビジネス電子版 | nikkeibiz |
| 67 | Magazine | Bunshun Online | 文春オンライン | bunshun |
| 68 | Magazine | Daily Shincho | デイリー新潮 | dailyshincho |
| 69 | Magazine | Marisol | Marisol | marisol |
| 70 | Magazine | SPUR.JP | SPUR.JP | spur |
| 71 | Magazine | with online | with online | withonline |
| 72 | Magazine | MORE | MORE | more |
| 73 | Book | Comic Seymour | コミックシーモア | comicseymour |
| 74 | Book | Mecha Comic | めちゃコミック | mechacomic |
| 75 | Book | Piccoma | ビッコマ | piccoma |
| 76 | Book | LINE Manga | LINEマンガ | linemanga |
| 77 | Book | Amazon Kindle | Amazon Kindle | amazonkindle |
| 78 | Book | Rakuten Kobo | 楽天Kobo | rakutenkobo |
| 79 | Calendar | Google Calendar | Google カレンダー | googlecalendar |
| 80 | Calendar | Yahoo! Calendar | Yahoo!カレンダー | yahoocalendar |
| 81 | Calendar | TimeTree | TimeTree | timetree |
| 82 | Hobby | DMM.com | DMM.com | dmmcom |
| 83 | Hobby | Tamahiyo net | たまひよnet | tamahiyo |
| 84 | Hobby | Slownet | Slownet(スローネット) | slownet |
| 85 | Hobby | Shumijin Club | 趣味人倶楽部（しゅみーとくらぶ） | shumijin |
| 86 | Hobby | Halmek 365 | ハルメク365 | halmek |
| 87 | Hobby | pixiv | pixiv（ピクシブ） | pixiv |
| 88 | Hobby | OZmall | OZmall | ozmall |
| 89 | Learning | Study Sapuri | スタディサプリ | studysap |
| 90 | Map | Yahoo! Transit / Transfer Guide | Yahoo!路線情報／Yahoo!乗換案内 | yahooroute |
| 91 | Map | Ekispert | 駅すぱあと | ekispa |
| 92 | Map | NAVITIME / Transfer NAVITIME | NAVITIME／乗換NAVITIME | navitime |
| 93 | Map | Google Maps | Google マップ | googlemap |
| 94 | Map | Yahoo! Maps | Yahoo!地図／Yahoo!マップ | yahoomap |
| 95 | Map | Mapion | 地図マピオン (Mapion) | mapion |
| 96 | Map | Jorudan | ジョルダン | jordan |
| 97 | Map | Ekitan | 駅探（ekitan） | ekitan |
| 98 | Point | Rakuten Super Point Screen | 楽天スーパーポイントスクリーン | rakutenscreen |
| 99 | Point | Ponta | Ponta（ポンタ） | ponta |
| 100 | Point | Moppy | モッピー | moppy |
| 101 | Point | EC Navi | ECナビ | ecnavi |
| 102 | Point | V Point Site (formerly T-Site) | Vポイントサイト（旧Tサイト） | vpoint |
| 103 | Point | d POINT CLUB | dポイントクラブ | dpoint |
| 104 | Beauty | Hot Pepper Beauty | ホットペーパービューティー | hotpepperbeauty |
| 105 | Beauty | Rakuten Beauty | 楽天ビューティ | rakutenbeauty |
| 106 | Finance | Moneytree | Moneytree（マネーツリー） | moneytree |
| 107 | Finance | Money Forward | マネーフォワード | mfoward |
| 108 | General e-commerce | Rakuten Ichiba | 楽天市場 | rakutenichiba |
| 109 | General e-commerce | Amazon | Amazon | amazon |
| 110 | General e-commerce | Yahoo! Shopping | Yahoo!ショッピング | yahooshopping |
| 111 | General e-commerce | ZOZOTOWN | ZOZOTOWN | zozo |
| 112 | General e-commerce | au PAY Market | au PAY マーケット | aupay |
| 113 | General e-commerce | Qoo10 | Qoo10 | qoo10 |
| 114 | General e-commerce | SHOPLIST | SHOPLIST(ショップリスト) | shoplist |
| 115 | General e-commerce | Rakuten Fashion | Rakuten Fashion | rakutenfashion |
| 116 | Electronics store | Yodobashi Camera (Yodobashi.com) | ヨドバシカメラ（ヨドバシ.com） | yodobashi |
| 117 | Electronics store | Bic Camera (biccamera.com) | ビックカメラ（ビックカメラ.com） | biccamera |
| 118 | Electronics store | Yamada Holdings (Yamada Webcom) | ヤマダホールディングス（ヤマダウェブコム） | yamada |
| 119 | Electronics store | Joshin (Joshin Web Shop) | 上新電機（Joshin webショップ） | joshin |
| 120 | Retail | Mitsukoshi Isetan | 三越伊勢丹 | mitsukoshi |
| 121 | Retail | AEON Net Supermarket | イオンネットスーパー | aeon |
| 122 | Retail | Ito-Yokado | イトーヨーカドー | itoyokado |
| 123 | Retail | Nitori (Nitori Net) | ニトリ（ニトリネット） | nitori |
| 124 | Retail | Don Quijote | ドン・キホーテ | donki |
| 125 | Retail | 7-Eleven | セブン‐イレブン | seven |
| 126 | Retail | Lawson | ローソン | lawson |
| 127 | Retail | FamilyMart | ファミリーマート | familymart |
| 128 | Manufacturer e-commerce | UNIQLO | ユニクロ | uniqlo |
| 129 | Manufacturer e-commerce | Onward (ONWARD CROSSET) | オンワード（ONWARD CROSSET） | onward |
| 130 | Manufacturer e-commerce | Iris Ohyama (Iris Plaza) | アイリスオーヤマ（アイリスプラザ） | iris |
| 131 | Online shopping | @cosme | ＠cosme | cosme |
| 132 | Online shopping | LOHACO | アクスル（LOHACO） | lohaco |
| 133 | Online shopping | Senshukai (Belle Maison) | 千趣会（ベルメゾン） | bellemaison |
| 134 | Online shopping | Japanet Takata / Tsunagaru Japanet | ジャパネットたかた／つながるジャパネット | japanet |
| 135 | Online shopping | Jupiter Shop Channel (Shop Channel) | ジュピターショップチャンネル（ショップチャンネル） | jupiter |
| 136 | Online shopping | Dinos Online Shop | dinos（ディノスオンラインショップ） | dinos |
| 137 | Online shopping | Belluna | ベルーナ | belluna |
| 138 | Online shopping | Oisix | Oisix（オイシックス） | oisix |
| 139 | Online shopping | QVC Japan | QVCジャパン | qvc |
| 140 | Flea market | Rakuma (formerly FRIL) | ラクマ（旧 FRIL（フリル）） | rakuma |
| 141 | Flea market | Mercari | メルカリ（mercari） | mercari |
| 142 | Flea market | Yahoo! Flea Market (formerly PayPay Flea Market) | Yahoo!フリマ（旧PayPayフリマ） | yahooflea |
| 143 | Flea market | Yahoo! Auctions | Yahoo!オークション | yahooauction |
| 144 | Flea market | Jimoty | ジモティー | jmt |
| 145 | Flea market | BUYMA | BUYMA | buyma |
| 146 | Game | GameWith | ゲームウィズ | gamewith |
| 147 | Game | GREE | GREE | gree |
| 148 | Game | Game8 | Game8（ゲームエイト） | gameeight |
| 149 | Cooking recipe | Cookpad | クックパッド | cookpad |
| 150 | Cooking recipe | Rakuten Recipe | 楽天レシピ | rakutenrecipe |
| 151 | Cooking recipe | Kurashiru | クラシル | klassil |
| 152 | Cooking recipe | DELISH KITCHEN | DELISH　KITCHEN | delish |
| 153 | Restaurants | Hot Pepper Gourmet | ホットペッパーグルメ | hotpeppergourmet |
| 154 | Restaurants | Retty | Retty | retty |
| 155 | Restaurants | Gurunavi | ぐるなび | gurunavi |
| 156 | Restaurants | Tabelog | 食べログ | tabelog |
| 157 | Restaurants | Hitotsara | ヒトサラ | hitosara |
| 158 | Food delivery | Uber Eats | Uber Eats | ubereats |
| 159 | Food delivery | Demae-can | 出前館 | demaekan |
| 160 | Travel | Rakuten Travel | 楽天トラベル | rakutentravel |
| 161 | Travel | JTB Official | JTB公式 | jtb |
| 162 | Travel | HIS | HIS | his |
| 163 | Travel | Yahoo! Travel | Yahoo!トラベル | yahootravel |
| 164 | Travel | Ikyu.com | 一休.com | ikyu |
| 165 | Travel | Jalan.net | じゃらんnet | jalan |
| 166 | Travel | Trivago | トリバゴ | trivago |
| 167 | Travel | Travelko | トラベルコ | travelco |
| 168 | Travel | Expedia | Expedia | expedia |
| 169 | Travel | Agoda | Agoda | agoda |
| 170 | Travel | Hotels.com | Hotels.com | hotels |
| 171 | Travel | Skyscanner | スカイスキャナー | skyscanner |
| 172 | Travel | Booking.com | Booking.com | booking |
| 173 | Travel | TripAdvisor | トリップアドバイザー | tripadvisor |
| 174 | Travel | Ikoyo | いこーよ | ikoyo |
| 175 | Travel | aumo | aumo | aumo |
| 176 | Travel | JAL | JAL | jal |
| 177 | Travel | ANA | ANA | ana |
| 178 | Weather | tenki.jp | tenki.jp | tenki |
| 179 | Weather | Weathernews | ウェザーニュース | weathernews |
| 180 | Weather | Yahoo! Weather & Disaster | Yahoo!天気・災害 | yahooweather |
