# Supplementary material for: Strategy for Hepatitis B and C Virus Testing Campaigns Through Web Services and Digital Advertising in Japan: Nationwide Cross-Sectional Study With Correspondence Analysis
Source: J Med Internet Res. 2026 Apr 2;28:e89585. doi: 10.2196/89585 (PMC13046096; doi:10.2196/89585)
Supplement: Multimedia Appendix 2 [file jmir-v28-e89585-s002.docx]

# Multimedia Appendix 2. List of 25 types of digital advertising

| No | Digital Advertising | Variable name (analysis code) |
| --- | --- | --- |
| 1 | Seven-Eleven in-store and storefront digital advertising | SevenEleven_InStoreAds |
| 2 | FamilyMart in-store and storefront digital advertising | FamilyMart_InStoreAds |
| 3 | Lawson in-store and storefront digital advertising | Lawson_InStoreAds |
| 4 | Other convenience store in-store and storefront digital advertising | OtherConvenience_InStoreAds |
| 5 | AEON in-store and storefront digital advertising | Aeon_InStoreAds |
| 6 | Ito-Yokado in-store and storefront digital advertising | ItoYokado_InStoreAds |
| 7 | Other shopping mall / supermarket in-store and storefront digital advertising | OtherSupermarket_InStoreAds |
| 8 | Welcia in-store and storefront digital advertising | Welcia_InStoreAds |
| 9 | Sugi Pharmacy in-store and storefront digital advertising | SugiDrug_InStoreAds |
| 10 | Tsuruha Drug in-store and storefront digital advertising | Tsuruha_InStoreAds |
| 11 | Matsumoto Kiyoshi in-store and storefront digital advertising | MatsumotoKiyoshi_InStoreAds |
| 12 | Drug Cosmos in-store and storefront digital advertising | DrugCosmos_InStoreAds |
| 13 | Cocokarafine in-store and storefront digital advertising | CocokaraFine_InStoreAds |
| 14 | Other drugstore in-store and storefront digital advertising | OtherDrugstore_InStoreAds |
| 15 | Don Quijote in-store and storefront digital advertising | DonQuijote_InStoreAds |
| 16 | Discount stores other than Don Quijote in-store and storefront digital advertising | OtherDiscountStore_InStoreAds |
| 17 | Digital advertising inside trains (electronic displays installed in train cars) | Train_Ads |
| 18 | Large outdoor vision screens on buildings and rooftops | OutdoorLargeScreen_Ads |
| 19 | Digital advertising on vending machines | VendingMachine_Ads |
| 20 | Digital advertising at stations and inside station facilities | Station_Ads |
| 21 | Digital advertising at bus stops / shelters | BusStop_Ads |
| 22 | Digital advertising inside taxis | Taxi_Ads |
| 23 | Other non-commercial facility digital advertising | OtherNonCommercial_Ads |
| 24 | Other digital advertising | OtherDigital_Ads |
| 25 | Nothing particular | None |
