# Supplementary material for: Strategy for Hepatitis B and C Virus Testing Campaigns Through Web Services and Digital Advertising in Japan: Nationwide Cross-Sectional Study With Correspondence Analysis
Source: J Med Internet Res. 2026 Apr 2;28:e89585. doi: 10.2196/89585 (PMC13046096; doi:10.2196/89585)
Supplement: Multimedia Appendix 3 [file jmir-v28-e89585-s003.docx]

# Multimedia Appendix 3. Variables used in modified Poisson regression models for digital exposure and desire for hepatitis virus testing

| (A) Web services using modified Poisson regression models | | | |
| --- | --- | --- | --- |
| Category | Variable | Data type | Coding / Range |
| Outcome | Use of each web service (amazonprime, abema, tver, youtube, radiko, facebook, instagram, line, x, google, yahoojapan, infoseek, googlenews, yahoonews, wikipedia, nikkei, googlecalendar, yahooroute, navitime, googlemap, yahoomap, rakutenscreen, ponta, moppy, ecnavi, vpoint, dpoint, hotpepperbeauty, rakutenichiba, amazon, yahooshopping, zozo, yodobashi, biccamera, nitori, seven, lawson, familymart, uniqlo, cosme, mercari, yahooauction, cookpad, rakutenrecipe, klassil, hotpeppergourmet, gurunavi, tabelog, rakutentravel, yahootravel, ikyu, jalan, jal, tenki, weathernews, yahooweather) | Categorical | 1 = selected; 0 = not selected |
| Main independent variable | Intention to undergo viral hepatitis testing | Categorical | 1 = “never having been tested and wishing to be tested”; 0 = all other response options |
| Covariate | Age | Continuous | 20–69 (years) |
| Covariate | Sex | Categorical | Male / Female (analysis coding as defined in dataset) |
| Covariate | Educational attainment | Categorical | 0 = below bachelor’s degree; 1 = bachelor’s degree or higher |
| Covariate | Marital status | Categorical | 0 = not currently married; 1 = currently married |
| Covariate | Household income (JPY, Japanese yen, $1=JPY159) | Continuous | 1 = ≤4.0 million JPY; 2 = 4.01–6.0 million JPY; 3 = 6.01–8.0 million JPY; 4 = 8.01–10.0 million JPY; 5 = 10.01–12.0 million JPY; 6 = 12.01–15.0 million JPY; 7 = ≥15.01 million JPY |
| Covariate | Total number of digital services selected | Continuous | 0–180 |
|  |  |  |  |
| (B) Digital advertising channels using modified Poisson regression models | | | |
| Category | Variable | Data type | Coding / Range |
| Outcome | Use of each digital advertising channel (SevenEleven_InStoreAds, FamilyMart_InStoreAds, Lawson_InStoreAds, OtherConvenience_InStoreAds, Aeon_InStoreAds, ItoYokado_InStoreAds, OtherSupermarket_InStoreAds, Welcia_InStoreAds, SugiDrug_InStoreAds, Tsuruha_InStoreAds, MatsumotoKiyoshi_InStoreAds, DrugCosmos_InStoreAds, CocokaraFine_InStoreAds, OtherDrugstore_InStoreAds, DonQuijote_InStoreAds, OtherDiscountStore_InStoreAds, Train_Ads, OutdoorLargeScreen_Ads) | Categorical | 1 = selected; 0 = not selected |
| Main independent variable | Intention to undergo viral hepatitis testing | Categorical | 1 = “never having been tested and wishing to be tested”; 0 = all other response options |
| Covariate | Age | Continuous | 20–69 (years) |
| Covariate | Sex | Categorical | Male / Female (analysis coding as defined in dataset) |
| Covariate | Educational attainment | Categorical | 0 = below bachelor’s degree; 1 = bachelor’s degree or higher |
| Covariate | Marital status | Categorical | 0 = not currently married; 1 = currently married |
| Covariate | Household income (JPY, Japanese yen, $1=JPY159) | Continuous | 1 = ≤4.0 million JPY; 2 = 4.01–6.0 million JPY; 3 = 6.01–8.0 million JPY; 4 = 8.01–10.0 million JPY; 5 = 10.01–12.0 million JPY; 6 = 12.01–15.0 million JPY; 7 = ≥15.01 million JPY |
| Covariate | Total number of digital advertising channels selected | Continuous | 0–180 |
