# Supplementary material for: Strategy for Hepatitis B and C Virus Testing Campaigns Through Web Services and Digital Advertising in Japan: Nationwide Cross-Sectional Study With Correspondence Analysis
Source: J Med Internet Res. 2026 Apr 2;28:e89585. doi: 10.2196/89585 (PMC13046096; doi:10.2196/89585)
Supplement: Multimedia Appendix 4 [file jmir-v28-e89585-s004.docx]

## Multimedia Appendix 4. Distribution of educational background in this study and the census 2020

|  | This study | |  | Census 2020 | |
| --- | --- | --- | --- | --- | --- |
|  | Males | Females |  | Males | Females |
| Middle school | 1.5 | 1.4 |  | 6.2 | 4.6 |
| High school or Vocational school | 33.8 | 41.2 |  | 34.5 | 35.9 |
| Junior college / Technical college | 2.7 | 16.5 |  | 9.1 | 24.1 |
| University | 50.1 | 34.8 |  | 27.8 | 17.9 |
| Graduate school | 6.1 | 2.8 |  | 3.8 | 1.3 |
| Currently enrolled | 4.6 | 2.6 |  | 3.3 | 2.9 |
| Other | 1.1 | 0.7 |  | 15.4 | 13.3 |
