# Supplementary figures and images for: Strategy for Hepatitis B and C Virus Testing Campaigns Through Web Services and Digital Advertising in Japan: Nationwide Cross-Sectional Study With Correspondence Analysis
Source: J Med Internet Res. 2026 Apr 2;28:e89585. doi: 10.2196/89585 (PMC13046096; doi:10.2196/89585)

# Multimedia Appendix 5. Distribution of household income in this study and the census 2020


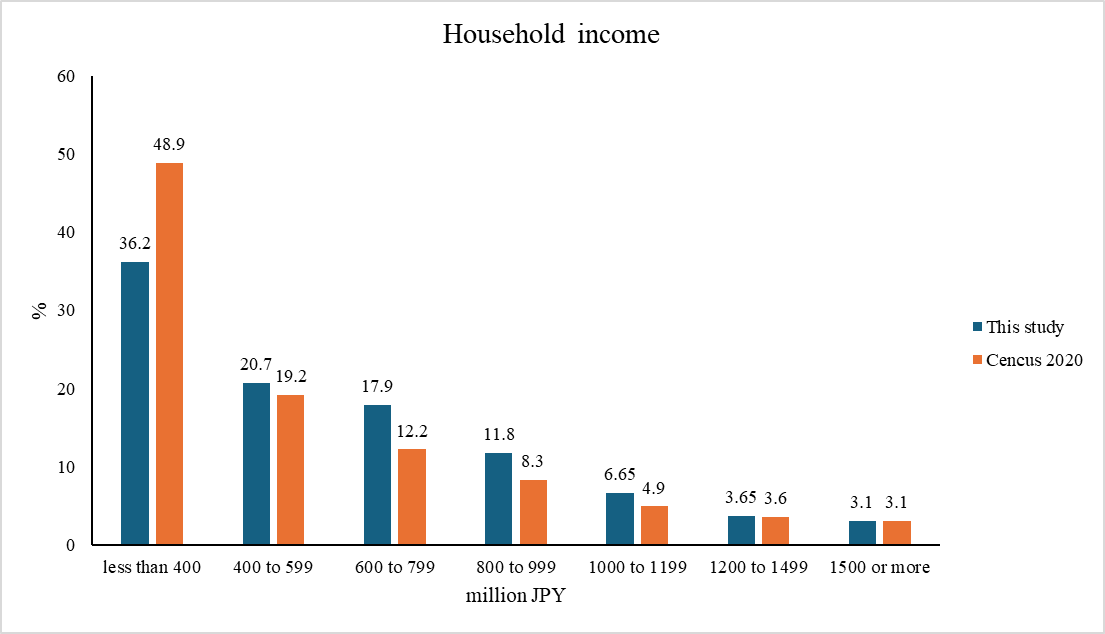

Supplement: Multimedia Appendix 5 [file jmir-v28-e89585-s005.docx]
