# Supplementary material for: Strategy for Hepatitis B and C Virus Testing Campaigns Through Web Services and Digital Advertising in Japan: Nationwide Cross-Sectional Study With Correspondence Analysis
Source: J Med Internet Res. 2026 Apr 2;28:e89585. doi: 10.2196/89585 (PMC13046096; doi:10.2196/89585)
Supplement: Multimedia Appendix 6 [file jmir-v28-e89585-s006.docx]

# Multimedia Appendix 6. Distribution of occupation and industry in this study and the census 2020

|  |  | This study | | | |  | Census 2020 | |
| --- | --- | --- | --- | --- | --- | --- | --- | --- |
|  |  | Males | | Females | |  | Males | Females |
|  |  | n | % | n | % |  | % | % |
| Occupation | |  |  |  |  |  |  |  |
|  | Not working | 165 | - | 306 | - |  | - | - |
|  | Administrative and managerial workers | 123 | 14.5 | 13 | 1.9 |  | 2.1 | 0.7 |
|  | Professional and engineering workers | 158 | 18.7 | 103 | 15.1 |  | 18.7 | 20.2 |
|  | Clerical workers | 138 | 16.3 | 224 | 32.8 |  | 21.0 | 28.3 |
|  | Sales workers | 49 | 5.8 | 55 | 8.1 |  | 12.4 | 12.5 |
|  | Service workers | 104 | 12.3 | 136 | 19.9 |  | 12.1 | 18.4 |
|  | Security workers | 8 | 0.9 | 0 | 0.0 |  | 2.0 | 0.3 |
|  | Agriculture, forestry and fishery workers | 11 | 1.3 | 2 | 0.3 |  | 3.1 | 2.5 |
|  | Manufacturing process workers | 53 | 6.3 | 27 | 4.0 |  | 13.4 | 8.8 |
|  | Transport and machine operation workers | 21 | 2.5 | 3 | 0.4 |  | 3.5 | 0.3 |
|  | Construction and mining workers | 25 | 3.0 | 5 | 0.7 |  | 4.4 | 0.3 |
|  | Carrying, cleaning, packaging, and related workers | 28 | 3.3 | 15 | 2.2 |  | 7.5 | 7.6 |
|  | Unclassified occupation | 128 | 15.1 | 100 | 14.6 |  |  |  |
| Industry | |  |  |  |  |  |  |  |
|  | Agriculture and forestry | 11 | 1.3 | 3 | 0.4 |  | 3.5 | 2.8 |
|  | Fishery | 0 | 0.0 | 0 | 0.0 |  | 0.3 | 0.1 |
|  | Mining and quarrying of stone and gravel | 2 | 0.2 | 0 | 0.0 |  | 0.1 | 0.0 |
|  | Construction | 66 | 7.8 | 28 | 4.1 |  | 11.1 | 2.9 |
|  | Manufacturing | 156 | 18.4 | 64 | 9.4 |  | 19.4 | 10.7 |
|  | Electricity, gas, heat supply and water | 13 | 1.5 | 7 | 1.0 |  | 0.7 | 0.2 |
|  | Information and communications | 62 | 7.3 | 24 | 3.5 |  | 4.4 | 2.2 |
|  | Transport and postal services | 54 | 6.4 | 18 | 2.6 |  | 7.7 | 2.6 |
|  | Wholesale and retail trade | 76 | 9.0 | 95 | 13.9 |  | 13.2 | 17.9 |
|  | Finance and insurance | 31 | 3.7 | 32 | 4.7 |  | 1.8 | 3.0 |
|  | Real estate and goods rental and leasing | 26 | 3.1 | 20 | 2.9 |  | 2.3 | 2.0 |
|  | Scientific research, professional and technical services | 32 | 3.8 | 16 | 2.3 |  | 4.3 | 3.0 |
|  | Accommodations, eating and drinking services | 24 | 2.8 | 38 | 5.6 |  | 3.7 | 7.5 |
|  | Living-related and personal services and amusement services | 16 | 1.9 | 31 | 4.5 |  | 2.5 | 4.6 |
|  | Education, learning support | 33 | 3.9 | 38 | 5.6 |  | 3.7 | 6.4 |
|  | Medical, health care and welfare | 60 | 7.1 | 120 | 17.6 |  | 5.9 | 22.1 |
|  | Compound services | 7 | 0.8 | 7 | 1.0 |  | 0.8 | 0.7 |
|  | Other services | 81 | 9.6 | 63 | 9.2 |  | 7.2 | 5.9 |
|  | Government | 44 | 5.2 | 32 | 4.7 |  | 4.5 | 2.4 |
|  | Unclassified industries | 52 | 6.1 | 47 | 6.9 |  | 2.8 | 3.2 |
