# Supplementary material for: Strategy for Hepatitis B and C Virus Testing Campaigns Through Web Services and Digital Advertising in Japan: Nationwide Cross-Sectional Study With Correspondence Analysis
Source: J Med Internet Res. 2026 Apr 2;28:e89585. doi: 10.2196/89585 (PMC13046096; doi:10.2196/89585)
Supplement: Multimedia Appendix 7 [file jmir-v28-e89585-s007.docx]

# Multimedia Appendix 7. Distribution of residential regions in this study and the 2020 Population Census

|  | (1) This study | |  | (2) Census 2020 | |  | Difference, (1)-(2) | |
| --- | --- | --- | --- | --- | --- | --- | --- | --- |
|  | (20-69 years old) | |  | (20-69 years old) | |  |  | |
|  | Males | Females |  | Males | Females |  | Males | Females |
|  | % | % |  | % | % |  | % | % |
| Hokkaido | 4.06 | 3.74 |  | 4.03 | 4.25 |  | 0.02 | -0.51 |
| Aomori | 0.89 | 0.61 |  | 0.95 | 0.98 |  | -0.06 | -0.37 |
| Iwate | 0.99 | 0.51 |  | 0.94 | 0.92 |  | 0.05 | -0.42 |
| Miyagi | 2.57 | 1.82 |  | 1.86 | 1.85 |  | 0.71 | -0.03 |
| Akita | 0.79 | 0.30 |  | 0.72 | 0.73 |  | 0.07 | -0.43 |
| Yamagata | 0.89 | 0.40 |  | 0.83 | 0.81 |  | 0.07 | -0.41 |
| Fukushima | 1.29 | 1.31 |  | 1.48 | 1.40 |  | -0.19 | -0.09 |
| Ibaraki | 1.19 | 1.42 |  | 2.34 | 2.21 |  | -1.16 | -0.79 |
| Tochigi | 1.19 | 0.81 |  | 1.60 | 1.50 |  | -0.41 | -0.69 |
| Gunma | 1.58 | 1.92 |  | 1.55 | 1.48 |  | 0.04 | 0.44 |
| Saitama | 5.34 | 6.17 |  | 6.02 | 5.80 |  | -0.68 | 0.37 |
| Chiba | 4.85 | 4.35 |  | 5.16 | 4.98 |  | -0.31 | -0.63 |
| Tokyo | 14.54 | 14.66 |  | 12.13 | 11.96 |  | 2.41 | 2.70 |
| Kanagawa | 9.99 | 9.91 |  | 7.76 | 7.44 |  | 2.24 | 2.47 |
| Niigata | 1.19 | 1.21 |  | 1.72 | 1.69 |  | -0.53 | -0.47 |
| Toyama | 1.09 | 0.91 |  | 0.80 | 0.78 |  | 0.29 | 0.13 |
| Ishikawa | 0.49 | 0.81 |  | 0.88 | 0.87 |  | -0.38 | -0.07 |
| Fukui | 0.69 | 0.51 |  | 0.60 | 0.59 |  | 0.10 | -0.08 |
| Yamanashi | 0.30 | 0.10 |  | 0.63 | 0.62 |  | -0.34 | -0.52 |
| Nagano | 1.29 | 1.62 |  | 1.56 | 1.53 |  | -0.28 | 0.09 |
| Gifu | 1.78 | 1.42 |  | 1.51 | 1.53 |  | 0.27 | -0.11 |
| Shizuoka | 2.57 | 3.34 |  | 2.92 | 2.80 |  | -0.35 | 0.53 |
| Aichi | 6.92 | 7.28 |  | 6.20 | 5.91 |  | 0.73 | 1.37 |
| Mie | 0.99 | 1.21 |  | 1.38 | 1.36 |  | -0.39 | -0.15 |
| Shiga | 0.79 | 0.91 |  | 1.12 | 1.11 |  | -0.33 | -0.20 |
| Kyoto | 1.78 | 3.34 |  | 1.94 | 2.04 |  | -0.16 | 1.30 |
| Osaka | 9.00 | 7.89 |  | 6.88 | 7.18 |  | 2.13 | 0.70 |
| Hyogo | 5.14 | 4.25 |  | 4.07 | 4.35 |  | 1.08 | -0.10 |
| Nara | 1.09 | 1.21 |  | 0.97 | 1.06 |  | 0.12 | 0.15 |
| Wakayama | 0.40 | 0.71 |  | 0.68 | 0.72 |  | -0.29 | -0.02 |
| Tottori | 0.79 | 0.20 |  | 0.42 | 0.42 |  | 0.37 | -0.22 |
| Shimane | 0.49 | 0.40 |  | 0.50 | 0.49 |  | -0.01 | -0.08 |
| Okayama | 1.48 | 1.82 |  | 1.39 | 1.43 |  | 0.09 | 0.39 |
| Hiroshima | 2.47 | 2.73 |  | 2.16 | 2.17 |  | 0.31 | 0.56 |
| Yamaguchi | 0.30 | 0.71 |  | 0.99 | 1.01 |  | -0.69 | -0.30 |
| Tokushima | 0.69 | 0.51 |  | 0.52 | 0.54 |  | 0.17 | -0.04 |
| Kagawa | 0.59 | 0.61 |  | 0.71 | 0.72 |  | -0.12 | -0.12 |
| Ehime | 0.79 | 0.91 |  | 0.98 | 1.02 |  | -0.18 | -0.11 |
| Kochi | 0.30 | 0.10 |  | 0.50 | 0.52 |  | -0.20 | -0.42 |
| Fukuoka | 3.56 | 3.74 |  | 3.86 | 4.13 |  | -0.30 | -0.39 |
| Saga | 0.49 | 0.40 |  | 0.60 | 0.63 |  | -0.11 | -0.23 |
| Nagasaki | 0.49 | 1.01 |  | 0.97 | 1.03 |  | -0.48 | -0.02 |
| Kumamoto | 0.69 | 0.61 |  | 1.28 | 1.36 |  | -0.59 | -0.75 |
| Oita | 0.30 | 0.61 |  | 0.82 | 0.86 |  | -0.53 | -0.25 |
| Miyazaki | 0.30 | 0.30 |  | 0.78 | 0.83 |  | -0.48 | -0.52 |
| Kagoshima | 0.30 | 0.61 |  | 1.14 | 1.23 |  | -0.84 | -0.62 |
| Okinawa | 0.30 | 0.10 |  | 1.18 | 1.18 |  | -0.88 | -1.08 |
