# Supplementary material for: Strategy for Hepatitis B and C Virus Testing Campaigns Through Web Services and Digital Advertising in Japan: Nationwide Cross-Sectional Study With Correspondence Analysis
Source: J Med Internet Res. 2026 Apr 2;28:e89585. doi: 10.2196/89585 (PMC13046096; doi:10.2196/89585)
Supplement: Multimedia Appendix 8 [file jmir-v28-e89585-s008.docx]

# Multimedia Appendix 8. The top-selected 50 web services

| Rank | Web service(description) | Variable name (analysis code) | Respondents selecting this option, n |
| --- | --- | --- | --- |
| 1 | Rakuten Ichiba | rakutenichiba | 863 |
| 2 | Google | google | 740 |
| 3 | Amazon | amazon | 690 |
| 4 | YouTube | youtube | 669 |
| 5 | Yahoo! Japan | yahoojapan | 658 |
| 6 | Yahoo! News | yahoonews | 624 |
| 7 | Google Maps | googlemap | 589 |
| 8 | Yahoo! Weather & Disaster | yahooweather | 503 |
| 9 | Rakuten Travel | rakutentravel | 455 |
| 10 | Tabelog | tabelog | 420 |
| 11 | Yahoo! Shopping | yahooshopping | 344 |
| 12 | Wikipedia | wikipedia | 331 |
| 13 | X (formerly Twitter) | x | 298 |
| 14 | Cookpad | cookpad | 294 |
| 15 | Yahoo! Transit / Route Finder | yahooroute | 286 |
| 16 | Hot Pepper Gourmet | hotpeppergourmet | 278 |
| 17 | Gurunavi | gurunavi | 277 |
| 18 | LINE | Line | 275 |
| 19 | Weathernews | weathernews | 264 |
| 20 | Rakuten Super Point Screen | rakutenscreen | 261 |
| 21 | UNIQLO | uniqlo | 251 |
| 22 | TVer | tver | 244 |
| 23 | Yodobashi Camera (yodobashi.com) | yodobashi | 244 |
| 24 | Hot Pepper Beauty | hotpepperbeauty | 239 |
| 25 | Google News | googlenews | 235 |
| 26 | Yahoo! Maps | yahoomap | 227 |
| 27 | EC Navi | ecnavi | 221 |
| 28 | Jalan.net | jalan | 203 |
| 29 | Ponta | ponta | 200 |
| 30 | Yahoo! Auctions | yahooauction | 200 |
| 31 | Instagram | instagram | 190 |
| 32 | Amazon Prime Video | amazonprime | 188 |
| 33 | d POINT Club | dpoint | 182 |
| 34 | Mercari | mercari | 182 |
| 35 | Google Calendar | googlecalendar | 173 |
| 36 | V Point Site (formerly T Site) | vpoint | 170 |
| 37 | Yahoo! Travel | yahootravel | 162 |
| 38 | tenki.jp | tenki | 157 |
| 39 | Kurashiru | klassil | 151 |
| 40 | Facebook | facebook | 150 |
| 41 | FamilyMart | familymart | 139 |
| 42 | Ikkyu.com | ikyu | 125 |
| 43 | ZOZOTOWN | zozo | 125 |
| 44 | BicCamera (biccamera.com) | biccamera | 123 |
| 45 | ABEMA | abema | 118 |
| 46 | Lawson | lawson | 118 |
| 47 | Nitori (Nitori Net) | nitori | 118 |
| 48 | Infoseek | infoseek | 111 |
| 49 | 7-Eleven | seven | 110 |
| 50 | Moppy | moppy | 109 |

Abbreviations: Variable names in the “Variable name” column correspond to those used in the regression models.
