# Supplementary material for: Strategy for Hepatitis B and C Virus Testing Campaigns Through Web Services and Digital Advertising in Japan: Nationwide Cross-Sectional Study With Correspondence Analysis
Source: J Med Internet Res. 2026 Apr 2;28:e89585. doi: 10.2196/89585 (PMC13046096; doi:10.2196/89585)
Supplement: Multimedia Appendix 9 [file jmir-v28-e89585-s009.docx]

# Multimedia Appendix 9. The selection of 25 digital advertising

| Rank | Digital advertising channel (description) | Variable name (analysis code) | Respondents selecting the option, n | Respondents selecting　the option, % |
| --- | --- | --- | --- | --- |
| 1 | Nothing particular | None | 1062 | 53.1 |
| 2 | FamilyMart in-store and storefront digital advertising | FamilyMart_InStoreAds | 365 | 18.3 |
| 3 | Digital advertising inside trains (electronic displays installed in train cars) | Train_Ads | 358 | 17.9 |
| 4 | Digital advertising at stations and inside station facilities | Station_Ads | 346 | 17.3 |
| 5 | Seven-Eleven in-store and storefront digital advertising | SevenEleven_InStoreAds | 322 | 16.1 |
| 6 | Large outdoor vision screens on buildings and rooftops | OutdoorLargeScreen_Ads | 299 | 15.0 |
| 7 | Lawson in-store and storefront digital advertising | Lawson_InStoreAds | 282 | 14.1 |
| 8 | AEON in-store and storefront digital advertising | Aeon_InStoreAds | 208 | 10.4 |
| 9 | Digital advertising on vending machines | VendingMachine_Ads | 160 | 8.0 |
| 10 | Digital advertising inside taxis | Taxi_Ads | 137 | 6.9 |
| 11 | Other non-commercial facility digital advertising | OtherNonCommercial_Ads | 115 | 5.8 |
| 12 | Don Quijote in-store and storefront digital advertising | DonQuijote_InStoreAds | 107 | 5.4 |
| 13 | Digital advertising at bus stops / shelters | BusStop_Ads | 101 | 5.1 |
| 14 | Other shopping mall / supermarket in-store and storefront digital advertising | OtherSupermarket_InStoreAds | 94 | 4.7 |
| 15 | Welcia in-store and storefront digital advertising | Welcia_InStoreAds | 86 | 4.3 |
| 16 | Matsumoto Kiyoshi in-store and storefront digital advertising | MatsumotoKiyoshi_InStoreAds | 86 | 4.3 |
| 17 | Ito-Yokado in-store and storefront digital advertising | ItoYokado_InStoreAds | 77 | 3.9 |
| 18 | Sugi Pharmacy in-store and storefront digital advertising | SugiDrug_InStoreAds | 75 | 3.8 |
| 19 | Other convenience store in-store and storefront digital advertising | OtherConvenience_InStoreAds | 69 | 3.5 |
| 20 | Tsuruha Drug in-store and storefront digital advertising | Tsuruha_InStoreAds | 63 | 3.2 |
| 21 | Drug Cosmos in-store and storefront digital advertising | DrugCosmos_InStoreAds | 61 | 3.1 |
| 22 | Other drugstore in-store and storefront digital advertising | OtherDrugstore_InStoreAds | 48 | 2.4 |
| 23 | Cocokarafine in-store and storefront digital advertising | CocokaraFine_InStoreAds | 47 | 2.4 |
| 24 | Discount stores other than Don Quijote in-store and storefront digital advertising | OtherDiscountStore_InStoreAds | 34 | 1.7 |
| 25 | Other digital advertising | OtherDigital_Ads | 32 | 1.6 |

Denominator: Percentages are based on all respondents (N=2000).

Multiple response: Respondents could select multiple options; therefore, percentages do not sum to 100%.

Abbreviations: Variable names in the “Variable name” column correspond to those used in the regression models.
