# Supplementary material for: Strategy for Hepatitis B and C Virus Testing Campaigns Through Web Services and Digital Advertising in Japan: Nationwide Cross-Sectional Study With Correspondence Analysis
Source: J Med Internet Res. 2026 Apr 2;28:e89585. doi: 10.2196/89585 (PMC13046096; doi:10.2196/89585)
Supplement: Multimedia Appendix 10 [file jmir-v28-e89585-s010.docx]

# Multimedia Appendix 10. Correspondence analysis between sex and web services


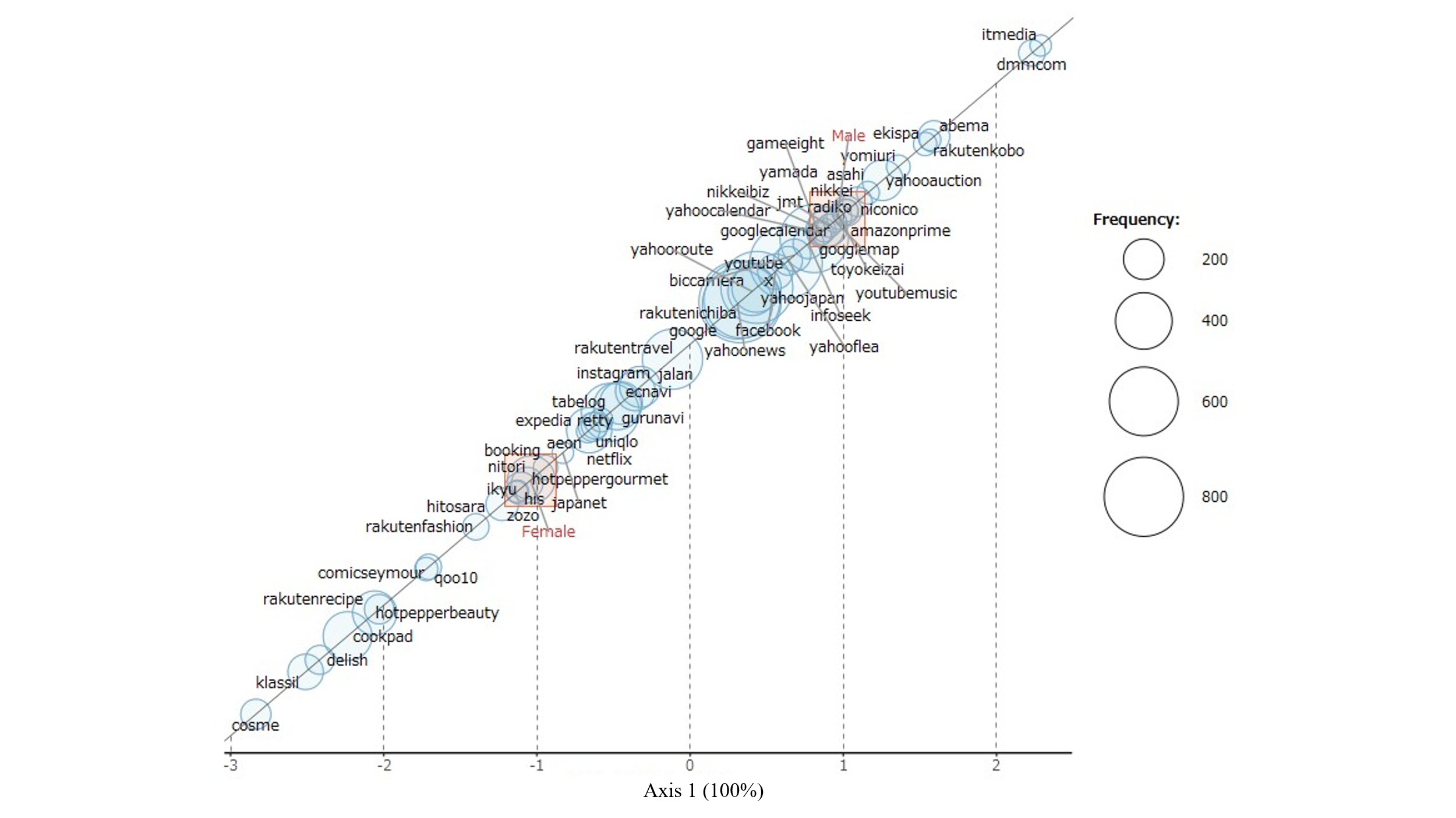


This figure presents a correspondence analysis of sex and web service use using all 2,000 respondents. The plot visualizes web services that were characteristically associated with males and females. In this analysis, services characteristically associated with males included internet technology services (IT media and DMM.com) and newspaper websites (Nikkei and Yomiuri), whereas services characteristically associated with females included cosmetic services and cooking recipe websites. Circle size indicates how many individuals selected each web service. Axis 1 explained 100% of the inertia in the sex-by–web service table, meaning that the male–female distribution contrast was fully captured on a single dimension.
