# Supplementary material for: Strategy for Hepatitis B and C Virus Testing Campaigns Through Web Services and Digital Advertising in Japan: Nationwide Cross-Sectional Study With Correspondence Analysis
Source: J Med Internet Res. 2026 Apr 2;28:e89585. doi: 10.2196/89585 (PMC13046096; doi:10.2196/89585)
Supplement: Multimedia Appendix 11 [file jmir-v28-e89585-s011.docx]

# Multimedia Appendix 11. Correspondence analysis between age groups and web services


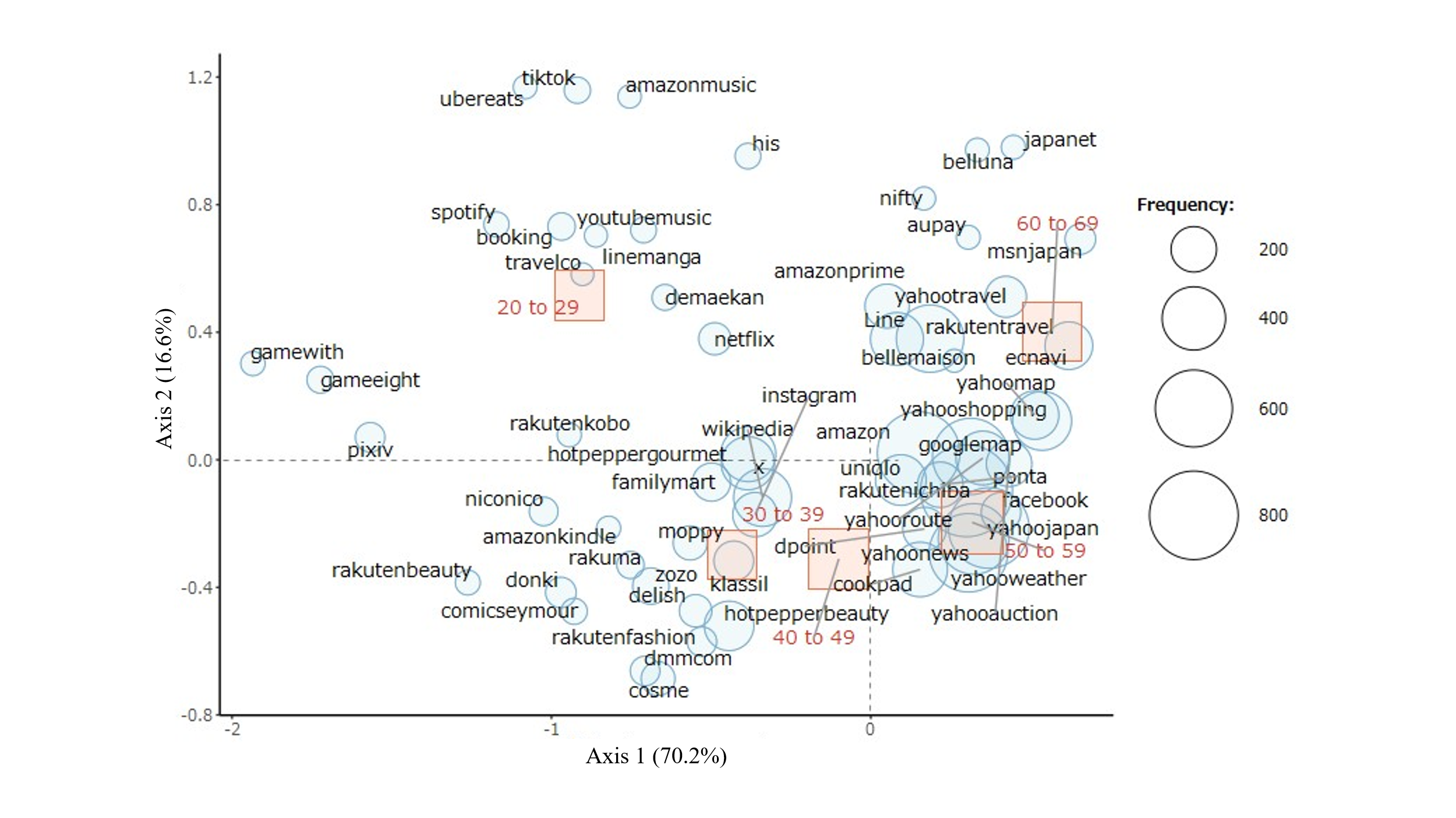


This figure presents a correspondence analysis of age group and web service use among all 2,000 respondents. The plot visualizes web services characteristically associated with each age group. In participants in their 20s, Uber Eats (food delivery), TikTok (video sharing), Spotify (music), and gaming services were prominent. In those in their 30s and 40s, prominent services included cosme (cosmetic shopping), Rakuten Fashion (general e-commerce), Hot Pepper Beauty (beauty salon booking and review site), and Cookpad (recipe sharing and cooking website). In respondents in their 50s, Yahoo-related services (search and related services) and Facebook were prominent, whereas in those in their 60s, Japanet (TV shopping), MSN Japan (online news/portal), and Belluna (mail-order and online shopping) were prominent. Circle size indicates the number of respondents who selected each web service. Axis 1 explained 70.2% of the inertia and Axis 2 explained 16.6%; together, they explained 86.8% of the total inertia, indicating that the two-dimensional map retained most of the information from the five age-group dimensions.
