# Supplementary material for: Strategy for Hepatitis B and C Virus Testing Campaigns Through Web Services and Digital Advertising in Japan: Nationwide Cross-Sectional Study With Correspondence Analysis
Source: J Med Internet Res. 2026 Apr 2;28:e89585. doi: 10.2196/89585 (PMC13046096; doi:10.2196/89585)
Supplement: Multimedia Appendix 12 [file jmir-v28-e89585-s012.docx]

# Multimedia Appendix 12. Sensitivity analysis of web services based on 3D correspondence analysis

| Ranking | Web service | Cos theta |
| --- | --- | --- |
| 1 | googlenews | 0.98 |
| 2 | zozo | 0.96 |
| 3 | nikkeibiz | 0.94 |
| 4 | comicseymour | 0.92 |
| 5 | lawson | 0.90 |
| 6 | yamada | 0.90 |
| 7 | joshin | 0.81 |
| 8 | demaekan | 0.79 |
| 9 | cosme | 0.77 |
| 10 | rakutenbeauty | 0.68 |


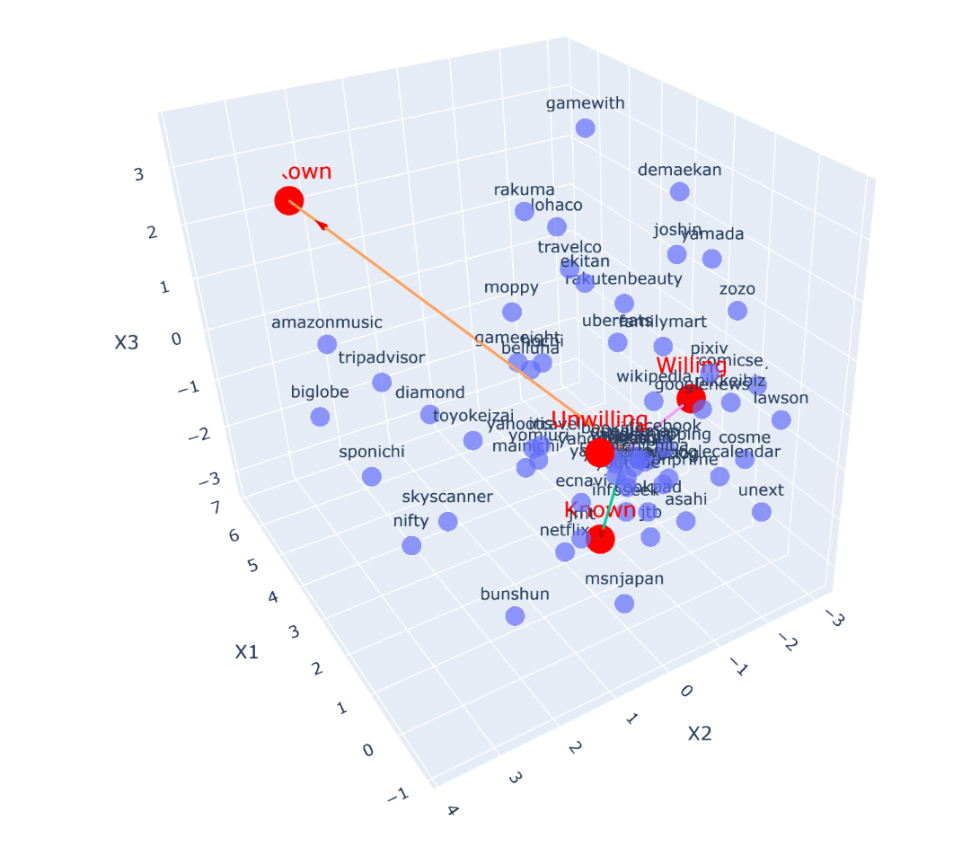


This figure presents a sensitivity analysis of web services using three-dimensional correspondence analysis. Web services characteristically associated with respondents who had not been tested and wanted to be tested were Google News (cosine θ=0.98), Zozotown (fashion e-commerce; cosine θ=0.96), Nikkei Biz (news media; cosine θ=0.94), Comic Seymour (e-book platform; cosine θ=0.92), and Lawson (convenience store; cosine θ=0.90). A cosine θ value closer to 1 indicates a stronger characteristic association with respondents who had not been tested and wanted to be tested.
