# Supplementary material for: Strategy for Hepatitis B and C Virus Testing Campaigns Through Web Services and Digital Advertising in Japan: Nationwide Cross-Sectional Study With Correspondence Analysis
Source: J Med Internet Res. 2026 Apr 2;28:e89585. doi: 10.2196/89585 (PMC13046096; doi:10.2196/89585)
Supplement: Multimedia Appendix 13 [file jmir-v28-e89585-s013.docx]

# Multimedia Appendix 13. Sensitivity analysis of digital advertising channels based on 3D correspondence analysis


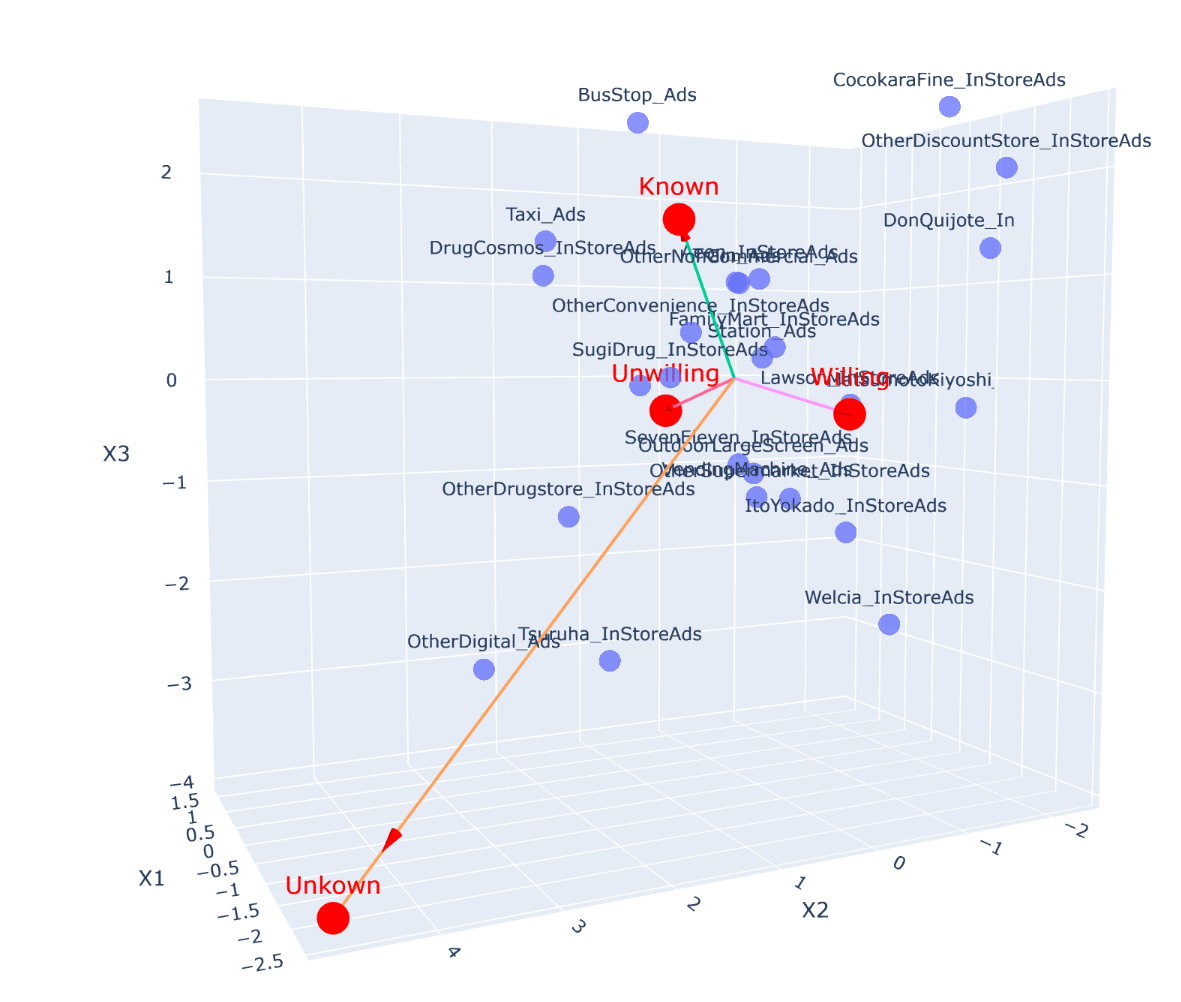


| Ranking | Digital advertising channels | Cos theta |
| --- | --- | --- |
| 1 | Lawson_InStoreAds | 0.96 |
| 2 | MatsumotoKiyoshi_InStoreAds | 0.92 |
| 3 | ItoYokado_InStoreAds | 0.78 |
| 4 | Welcia_InStoreAds | 0.77 |
| 5 | DonQuijote_InStoreAds | 0.69 |
| 6 | OtherSupermarket_InStoreAds | 0.66 |
| 7 | OtherDiscountStore_InStoreAds | 0.59 |
| 8 | VendingMachine_Ads | 0.50 |
| 9 | OutdoorLargeScreen_Ads | 0.44 |
| 10 | CocokaraFine_InStoreAds | 0.40 |

This figure presents a sensitivity analysis of digital advertising channels using 3-dimensional correspondence analysis. Digital advertising channels characteristically associated with respondents who had not been tested and wanted to be tested were Lawson in-store/storefront advertisements (cosine θ=0.96) and Matsumoto Kiyoshi in-store/storefront advertisements (cosine θ=0.92). A cosine θ value closer to 1 indicates a stronger characteristic association with respondents who had not been tested and wanted to be tested.
