# Supplementary material for: Strategy for Hepatitis B and C Virus Testing Campaigns Through Web Services and Digital Advertising in Japan: Nationwide Cross-Sectional Study With Correspondence Analysis
Source: J Med Internet Res. 2026 Apr 2;28:e89585. doi: 10.2196/89585 (PMC13046096; doi:10.2196/89585)
Supplement: Multimedia Appendix 14 [file jmir-v28-e89585-s014.docx]

# Multimedia Appendix 14. Associations between desire for hepatitis virus testing and use of individual web services


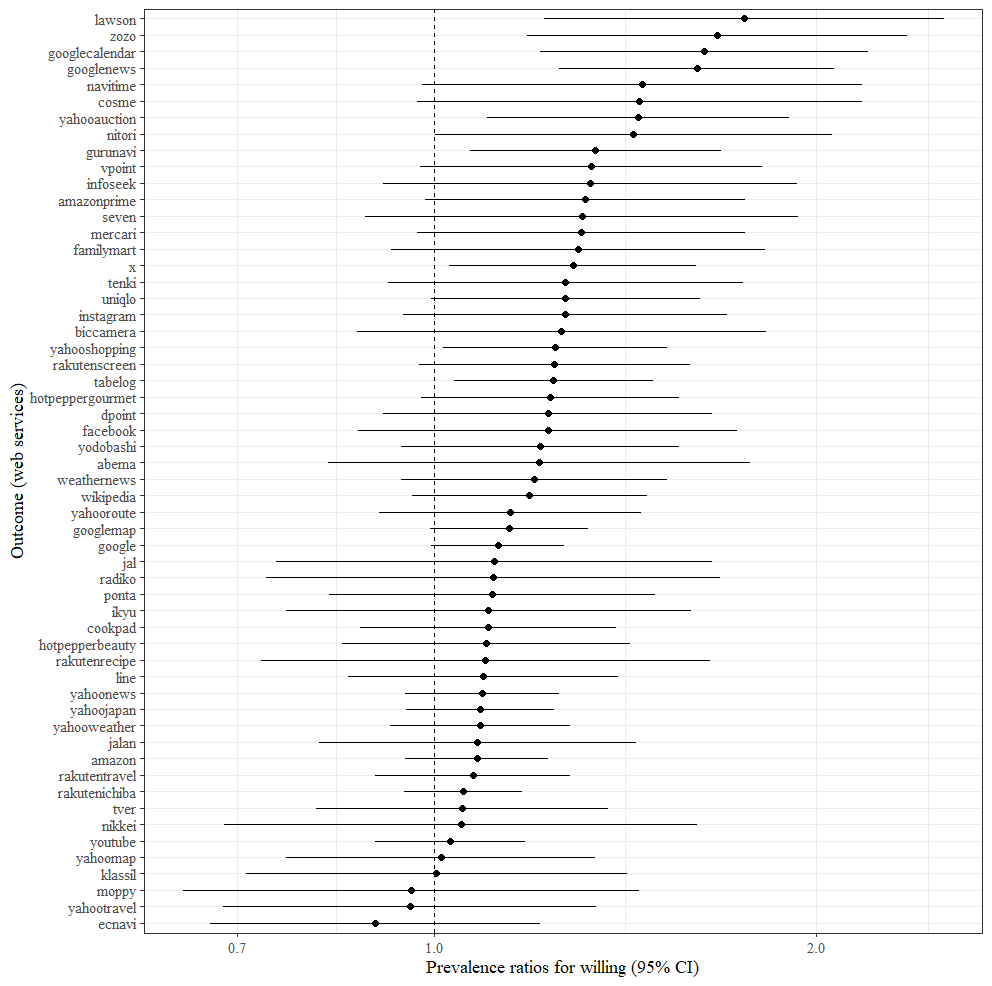


This presents sensitivity analyses of associations between desire for hepatitis virus testing and use of individual web services using modified Poisson regression. The highest prevalence ratios (PR) for desire for hepatitis virus testing were observed for Lawson (PR 1.75, 95% confidence interval [CI] 1.22-2.52), Zozotown (PR 1.67, 95% CI 1.18-2.36), Google Calendar (PR 1.63, 95% CI 1.21-2.20), and Google News (PR 1.61, 95% CI 1.25-2.07).
