# Supplementary material for: Strategy for Hepatitis B and C Virus Testing Campaigns Through Web Services and Digital Advertising in Japan: Nationwide Cross-Sectional Study With Correspondence Analysis
Source: J Med Internet Res. 2026 Apr 2;28:e89585. doi: 10.2196/89585 (PMC13046096; doi:10.2196/89585)
Supplement: Multimedia Appendix 15 [file jmir-v28-e89585-s015.docx]

# Multimedia Appendix 15. Associations between desire for hepatitis virus testing and exposure to individual digital advertising channels


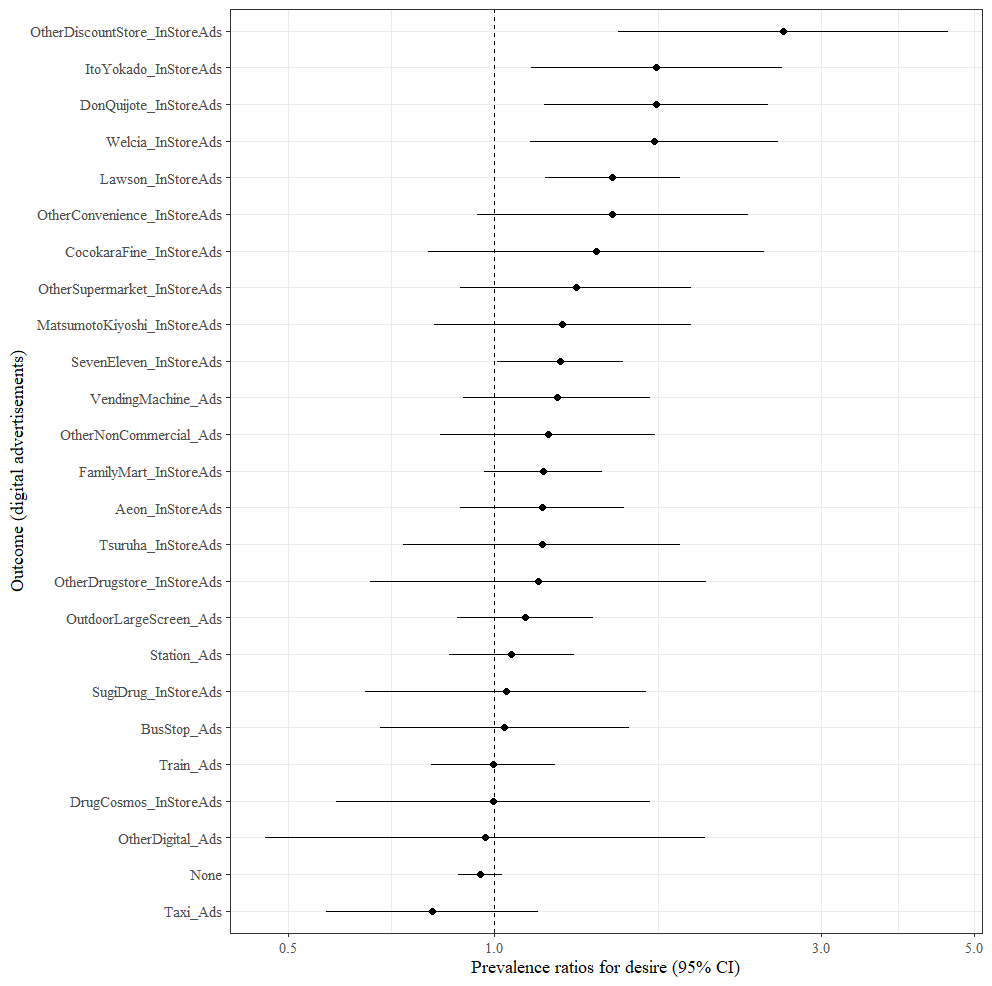


This presents sensitivity analyses of associations between desire for hepatitis virus testing and exposure to individual digital advertising channels using modified Poisson regression. The highest prevalence ratios (PR) were observed for other discount store in-store advertisements (PR 2.64, 95% confidence interval [CI] 1.51-4.59), Ito Yokado in-store advertisements (PR 1.72, 95% CI 1.13-2.63), Don Quijote in-store advertisements (PR 1.72, 95% CI 1.18-2.51), Welcia in-store advertisements (PR 1.71, 95% CI 1.13-2.59), and Lawson in-store advertisements (PR 1.49, 95% CI 1.19-1.87).
